# Metabolomic Analysis of Fermented Nori Powders: Divergence of Betaine Structural Analogs Production by Three Types of *koji* Fungal Fermentation

**DOI:** 10.3390/molecules30204104

**Published:** 2025-10-16

**Authors:** Nao Inoue, Konoka Kubo, Keisuke Tsuge, Ryosuke Sasaki, Akira Oikawa, Masatoshi Goto, Teruyoshi Yanagita, Koji Nagao

**Affiliations:** 1 Department of Biological Resource Science, Saga University, 1 Honjo-machi, Saga 840-8502, Japan; d5589@cc.saga-u.ac.jp (N.I.); mgoto@cc.saga-u.ac.jp (M.G.); yanagitt@cc.saga-u.ac.jp (T.Y.); 2 The United Graduate School of Agricultural Sciences, Kagoshima University, Kagoshima 890-0065, Japan; 3 Saga Regional Industry Support Center, Saga 849-0932, Japan; tsuge@saga-itc.jp; 4 RIKEN Center for Sustainable Resource Science, Yokohama 230-0045, Japan; ryosuke.sasaki@riken.jp (R.S.); oikawa.akira.7j@kyoto-u.ac.jp (A.O.); 5 Graduate School of Agriculture, Kyoto University, Kyoto 606-8502, Japan

**Keywords:** betaine, stachydrine, carnitine, *Aspergillus luchuensis* mut. *kawachii*, *Aspergillus oryzae*, *Monascus purpureus*, *Pyropia yezoensis*

## Abstract

Fermenting seaweed with *koji* fungi transforms its chemical composition, generating bioactive compounds absent in the raw material. We previously reported that the fungal fermentation of the edible red alga *Pyropia yezoensis* (Nori) produces betaine structural analogs (such as betaine, stachydrine, and carnitine), which are of particular interest because of their physiological roles and potential health benefits. Using metabolomic profiling, we compared non-fermented Nori with powders fermented by three industrially important fungi: *Aspergillus luchuensis* mut. *kawachii* (white *koji* fungus)*, Aspergillus oryzae* (yellow *koji* fungus)*,* and *Monascus purpureus* (red *koji* fungus). All fermentations enhanced the levels of betaine and carnitine, but stachydrine production was unique to the yellow *koji* fungus. Precursor patterns revealed distinct metabolic strategies: the yellow *koji* fungus exhibited an intermediate detectable choline oxidation route and strong proline methylation, the white *koji* fungus rapidly converted choline without intermediate accumulation, and the red *koji* fungus favored carnitine and proline but produced little stachydrine. Fermentation also shifted the methylation balance toward a state that supports methyl-dependent pathways. These findings reveal clear species-specific strategies for the production of betaine structural analogs, providing a mechanistic basis for understanding the metabolic divergence among *koji* fungi and guiding the targeted design of functional seaweed products.

## 1. Introduction

*Koji* fungi—*Aspergillus luchuensis* mut. *kawachii (A. kawachii)*, *Aspergillus oryzae (A. oryzae)*, and *Monascus purpureus (M. purpureus)*—are integral to traditional Japanese and East Asian fermentation techniques. Each species exhibits distinct enzymatic and metabolic activities that influence substrate transformation during the fermentation process. *A. kawachii*, known as white *koji* fungus, is primarily utilized in shochu production and is well known for its high citric acid production, which acidifies the fermentation environment, suppresses microbial contaminants, and contributes to antioxidant properties [1,2]. *A. oryzae*, known as the yellow *koji* fungus, is extensively used in the production of sake, miso, and soy sauce. It is characterized by robust secretion of hydrolytic enzymes, such as amylases and proteases, and its ability to produce large amounts of proteins and bioactive metabolites, making it a valuable biotechnological tool for food, pharmaceutical, and industrial applications [3,4]. *M. purpureus*, known as red *koji* fungus, is traditionally used in Chinese and Okinawan fermented foods and is notable for the generation of red pigments and bioactive compounds such as monacolin K, which possess cholesterol-lowering and antioxidant effects [5,6].

Our previous research demonstrated that fermentation of “Nori”, a sheet of edible red algae *Pyropia yezoensis* used in Japanese cuisine to prepare sushi and rice balls, with *A. oryzae* markedly produced three kinds of betaine structural analogs—namely betaine, stachydrine, and carnitine [7,8]. These compounds are of particular interest because of their diverse physiological roles and potential health benefits. Betaine, a glycine betaine, serves as an osmoprotectant and methyl group donor, playing critical roles in homocysteine metabolism and liver function, and is associated with improved physical performance and metabolic resilience [9,10]. Stachydrine, a proline betaine found in various plant and marine resources, has demonstrated anti-inflammatory, cardioprotective, and neuroprotective properties, making it a promising compound for functional food applications targeting vascular and cognitive health [11,12]. Carnitine, a lysine betaine, is essential for the mitochondrial transport of long-chain fatty acids, which are crucial for cellular energy production. Its supplementation may be beneficial for treating obesity, improving glucose intolerance, and increasing total energy expenditure [13,14].

This study aimed to perform a metabolomic analysis of Nori powders fermented with three types of *koji* fungi, focusing on the production of betaine structural analogs. By comparing the metabolite profiles of Nori powders fermented with each fungus, this study aimed to elucidate the fungus-specific pathways involved in the differential generation of betaine-related compounds. Understanding how each *koji* fungus influences the formation of these functional metabolites will not only provide insight into the bioconversion mechanism of marine algal substrates but also support the development of novel fermented seaweed-based functional foods with targeted health applications.

## 2. Results

### 2.1. Metabolomic Changes in Nori Powder During Fungal Fermentation

**Figure 1:**
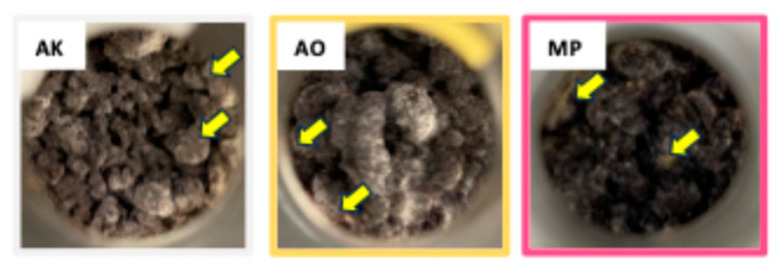
Appearance of Nori powder after 72 h of fermentation by *Aspergillus luchuensis* mut. *kawachii* (AK)*, Aspergillus oryzae* (AO), *and Monascus purpureus* (MP). The color of the photo frame indicates the color name of each *koji*. Yellow arrows indicate the representative spore-forming areas.

Our previous research has shown that betaine structural analog production begins during the late phase of microbial growth and is closely associated with spore formation [8]. As shown in Figure 1, after 72 h of *koji* fermentation, the surface of each Nori powder was covered with mycelium, and spore formation accompanied by partial color changes was observed.

**Figure 2:**
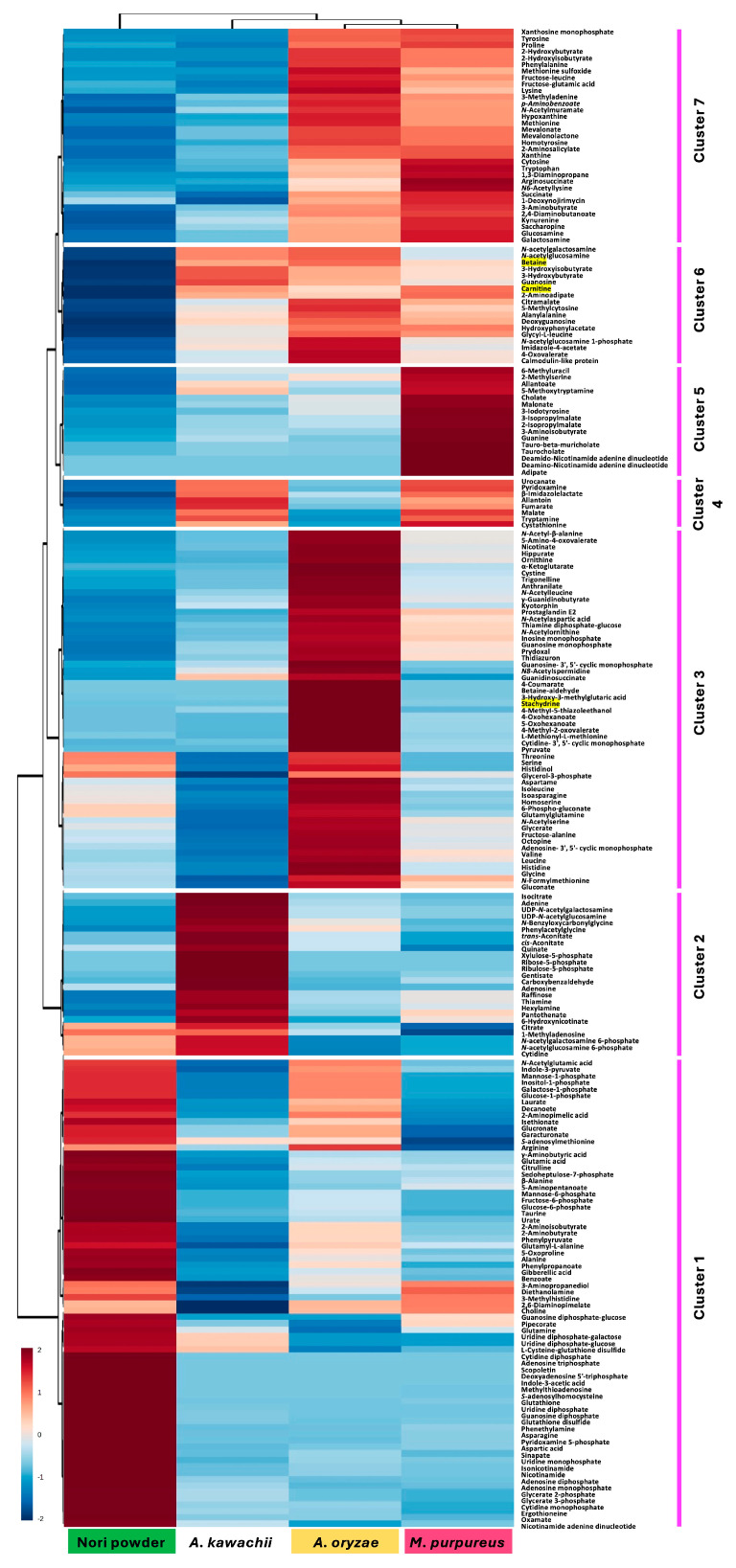
Hierarchical cluster analysis heatmap of 227 metabolites altered by 72 h *koji* fungal fermentation of Nori powder using three different fungi. Red and blue colors correspond to high and low relative metabolite levels, respectively.

Further, we performed metabolome analysis of water-soluble components to investigate differences in metabolites when Nori powder (NP) is fermented with three types of *koji* fungi. A total of 227 metabolites were identified and quantified using capillary electrophoresis time-of-flight mass spectrometry (CE-TOF MS), and changes in the metabolome of fermented NP were classified using hierarchical cluster analysis (HCA) (Figure 2).

The HCA heatmap revealed that the group of molecules commonly decreased across the three *koji* fermentations was classified into Cluster 1, and most of these compounds were associated with the degradation of cell wall polysaccharides of the red alga (Nori), including galactose-based sugars and their metabolic intermediates, such as UDP-galactose (UDP-Gal), UDP-glucose (UDP-Glc), and galactose-1-phosphate (Gal1P). In addition, urate, suggesting purine utilization during fermentation, and sulfur-containing compounds (e.g., taurine) were also included. Their consistent reduction compared to the unfermented NP indicates that the *koji* fungi actively degraded and assimilated these components as sources of carbon, nitrogen, and sulfur.

The molecules that increased characteristically in AK (white *koji* fungus) were classified into Cluster 2, including xylulose-5-phosphate, ribose-5-phosphate, and ribulose-5-phosphate (indicating activation of the pentose phosphate pathway), UDP-*N*-acetylglucosamine (UDP-GlcNAc) and UDP-*N*-acetylgalactosamine (UDP-GalNAc) (hexosamine pathway), citrate, cis- and trans-aconitate, and isocitrate (TCA cycle with citric acid accumulation), gentisate and quinate (aromatic compound degradation), 6-hydroxynicotinate (nicotinic acid degradation), and adenosine and adenine (nucleotide metabolism). These changes likely represent a metabolic adaptation that enables the white *koji* fungus to create an acidic environment and efficiently decompose and utilize sugars and aromatic components derived from seaweed powder.

The group of molecules that increased characteristically in AO (yellow *koji* fungus) was classified into Cluster 3, including amino acids derived from protein degradation such as valine, leucine, and isoleucine (branched-chain amino acids, BCAAs), organic acids like α-ketoglutarate and pyruvate related to the TCA cycle and carbohydrate metabolism, nucleotides such as inosine monophosphate (IMP) and guanosine monophosphate (GMP), coenzyme-related compounds like nicotinate and pyridoxal, as well as unique metabolites such as stachydrine and trigonelline. These features suggest the activation of amino acid metabolism (e.g., BCAA pathways), energy metabolism via the TCA cycle, nucleotide and vitamin pathways, and specialized metabolic routes utilizing seaweed-derived components.

The group of molecules that increased characteristically in MP (red *koji* fungus) was classified into Cluster 5, suggesting the activation of metabolic pathways related to nucleotide metabolism (guanine, 6-methyluracil, deamido-NAD, deamino-NAD), tryptophan metabolism (5-methoxytryptamine), BCAA biosynthesis (2- and 3-isopropylmalate), and lipid/bile acid-like compounds (cholate, taurocholate derivatives). In addition, the increase in malonate and adipate indicates possible alterations in organic acid metabolism and TCA cycle-related pathways. These findings suggest that the red *koji* fungus may enhance energy metabolism, secondary metabolism (such as polyketide and pigment synthesis), and amino acid and nucleotide biosynthesis.

The group of molecules that commonly increased during fermentation with the three types of *koji* fungi was classified into Cluster 6, and a notable feature was the significant production of betaine and carnitine, which are structural analogs of betaine, as well as allantoin. These compounds are involved in methyl-group transfer, osmotic regulation, lipid metabolism, and nitrogen recycling, suggesting the activation of stress response and energy metabolism during fermentation. In addition, increases in nucleotides such as guanosine and 5-methylcytosine, dipeptides such as Ala-Ala and Gly-Leu, and sugar derivatives such as GlcNAc and GalNAc indicate enhanced activity in nucleotide metabolism, amino acid catabolism, and cell wall-related carbohydrate metabolism.

**Figure 3:**
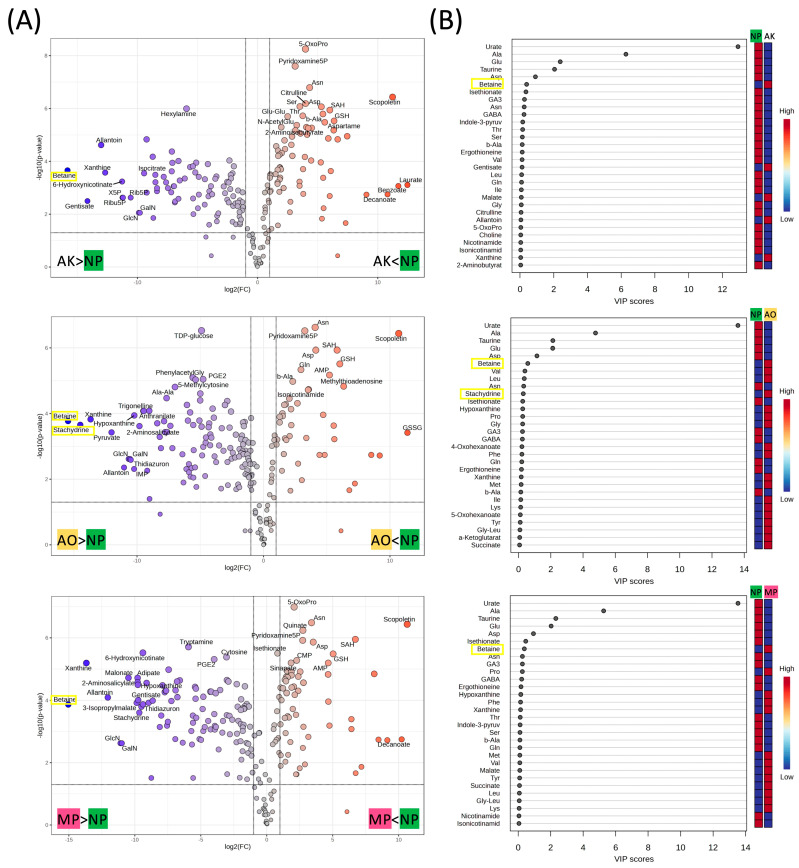
(**A**) Volcano plot of annotated metabolites significantly altered in each *koji* fungal fermented powder compared to unfermented Nori powder. (Thresholds used: |log2FC| > 1 and *p* < 0.05). The top 30 most significant metabolites (by *p*-value and/or fold change) are annotated with labels on the plot. (**B**) Partial least squares-discriminant analysis (PLS-DA) of powders fermented with each type of *koji* fungus versus unfermented Nori powder. Variable importance in projection (VIP) score plots of the top 30 PLS-DA metabolites are shown.

As shown in Figure 3A, volcano plot analysis comparing each *koji*-fermented sample (AK, AO, MP) with the original powder NP revealed numerous metabolites with significant fold changes and statistical relevance. Among the top 30 metabolites determined by the combined criterion of statistical significance (*y*-axis) and magnitude of change (*x*-axis) in the volcano plots, betaine appeared in all fermentation types, whereas stachydrine was included only in AO.

Furthermore, partial least squares discriminant analysis (PLS-DA) identified a clear separation between NP and each fermented group (Figure 3B). Among the top 30 variables with the highest variable importance in projection (VIP) scores, betaine consistently appeared across all fermentation types, whereas stachydrine was included among the top variables for AO.

Table 1 lists the top 30 metabolites (the complete list of quantified metabolites is provided in Tables S1–S3, and Venn diagrams of the top 30 metabolites with the greatest changes are shown in Figure S1) showing the greatest quantitative increases (µmol/g) in the three *koji*-fermented samples (AK, AO, MP) compared with the original powder NP. 

Among these, betaine exhibited the largest increase under all fermentation conditions, with +2.44 µmol/g in AK, +2.75 µmol/g in AO, and +2.14 µmol/g in MP. Stachydrine appeared prominently in AO, where it showed a marked increase of +1.41 µmol/g. In addition, carnitine ranked among the top metabolites in all fermented samples, increasing by +0.204 µmol/g in AK, +0.165 µmol/g in AO, and +0.224 µmol/g in MP. These findings indicate that *koji* fermentation strongly promotes the production of quaternary amine compounds such as betaine, stachydrine, and carnitine.

### 2.2. Differences in Betaine Production and Related Metabolite Levels

**Figure 4:**
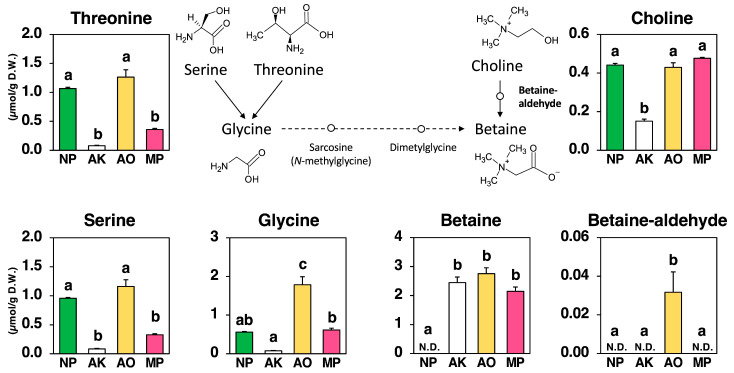
Betaine biosynthetic pathway and amounts of threonine, serine, glycine, betaine, betaine-aldehyde, and choline in non-fermented Nori (NP) and powders fermented with *A. luchuensis* mut. *kawachii* (AK), *Aspergillus oryzae* (AO), and *Monascus purpureus* (MP). Values are expressed as the mean ± standard error (n = 3 as biological replicates). N.D.: not detected. Different letters on the bars denote significant differences (*p* < 0.05) among the groups.

As shown in Figure 4, betaine, known as trimethylammonioacetate, was absent in NP but accumulated strongly in all fermented groups: AK (2.445 µmol/g), AO (2.754 µmol/g), and MP (2.142 µmol/g), confirming its de novo synthesis during fermentation.

Fermentation markedly altered the pools of choline and related metabolites. Choline levels remained relatively high in NP (0.441 µmol/g) and MP (0.476 µmol/g), moderate in AO (0.429 µmol/g), but dropped sharply in AK (0.151 µmol/g), suggesting rapid choline utilization in AK. The intermediate betaine-aldehyde was detected exclusively in AO (0.032 µmol/g), while remaining undetectable in NP, AK, and MP, indicating that AO exhibits a visible choline oxidation route, whereas AK and MP likely convert choline to betaine without intermediate accumulation.

Among the amino acid precursors, glycine increased markedly in AO (1.784 µmol/g) compared to NP (0.561 µmol/g), remained moderate in MP (0.615 µmol/g), and was lowest in AK (0.077 µmol/g). Similarly, serine and threonine—both potential glycine donors—rose in AO (Serine 1.159, Threonine 1.264 µmol/g) relative to NP (Serine 0.956, Threonine 1.066 µmol/g), but declined in AK (Serine 0.083, Threonine 0.079 µmol/g) and MP (Serine 0.328, Threonine 0.358 µmol/g).

These patterns suggest that AO not only activates the choline oxidation pathway but also expands the glycine/serine/threonine pools, whereas AK relies on rapid choline-to-betaine conversion with minimal glycine involvement, and MP maintains intermediate levels of both choline and glycine-related precursors.

### 2.3. Differences in Stachydrine Production and Related Metabolite Level

**Figure 5:**
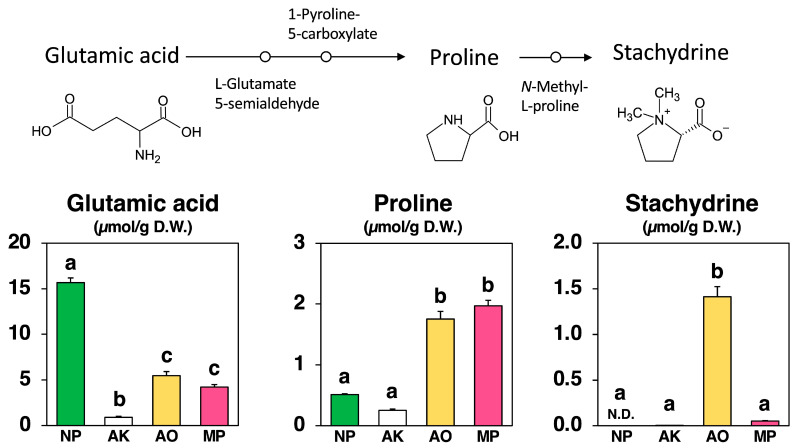
Stachydrine biosynthetic pathway and amounts of glutamic acid, proline, and stachydrine in non-fermented Nori (NP) and powders fermented with *A. luchuensis* mut. *kawachii* (AK), *Aspergillus oryzae* (AO), and *Monascus purpureus* (MP). Values are expressed as the mean ± standard error (n = 3 as biological replicates). N.D.: not detected. Different letters on the bars denote significant differences (*p* < 0.05) among the groups.

As shown in Figure 5, stachydrine, also known as (2S)-1,1-dimethylpyrrolidinium-2-carboxylate, which is derived by methylating the amino acid proline, was not detected in the Nori powder prior to fungal fermentation. Stachydrine, however, appeared after fermentation in a fungus-specific manner: *A. oryzae* (AO) produced 1.41 µmol/g, *M. purpureus* (MP) 0.0513 µmol/g, and *A. kawachii* (AK) only 0.00146 µmol/g.

Proline, the precursor amino acid of stachydrine, was present in NP at 0.513 µmol/g and increased markedly in AO (1.75 µmol/g) and MP (1.97 µmol/g), whereas AK showed a decrease to 0.253 µmol/g. Upstream, glutamate—the original substrate—was abundant in NP (15.67 µmol/g) but declined after fermentation to 5.47 µmol/g in AO, 4.22 µmol/g in MP, and 0.900 µmol/g in AK.

These results demonstrate that AO not only retained proline but also efficiently converted it to stachydrine, whereas AK exhibited strong depletion of both glutamate and proline with negligible stachydrine formation.

### 2.4. Differences in Carnitine Production and Related Metabolite Levels

**Figure 6:**
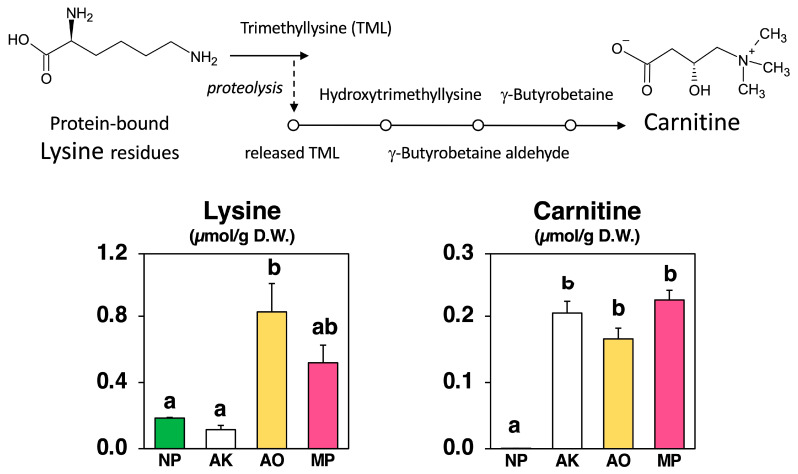
Carnitine biosynthetic pathway and amounts of lysine and carnitine in non-fermented Nori (NP) and powders fermented with *A. luchuensis* mut. *kawachii* (AK), *Aspergillus oryzae* (AO), and *Monascus purpureus* (MP). Values are expressed as the mean ± standard error (n = 3 as biological replicates). Different letters on the bars denote significant differences (*p* < 0.05) among the groups.

As shown in Figure 6, carnitine, also known as γ-trimethyl-β-oxybutyrobetain, which is derived by methylating the amino acid lysine, was slightly detected (0.00056 µmol/g) in Nori powder prior to fungal fermentation. Carnitine levels increased consistently across all fermented groups relative to NP, reaching 0.205 µmol/g in AK, 0.166 µmol/g in AO, and 0.224 µmol/g in MP.

In parallel, lysine concentrations exhibited marked strain-specific variation: AO showed the highest lysine (0.825 µmol/g), MP was intermediate (0.519 µmol/g), and AK had the lowest level (0.115 µmol/g), while NP remained relatively low (0.185 µmol/g).

These patterns suggest that carnitine accumulation is not directly linked to free lysine concentration, supporting the role of post-translational trimethyllysine (TML) generation as a key upstream step.

### 2.5. Differences in Methylation Potentials

**Figure 7:**
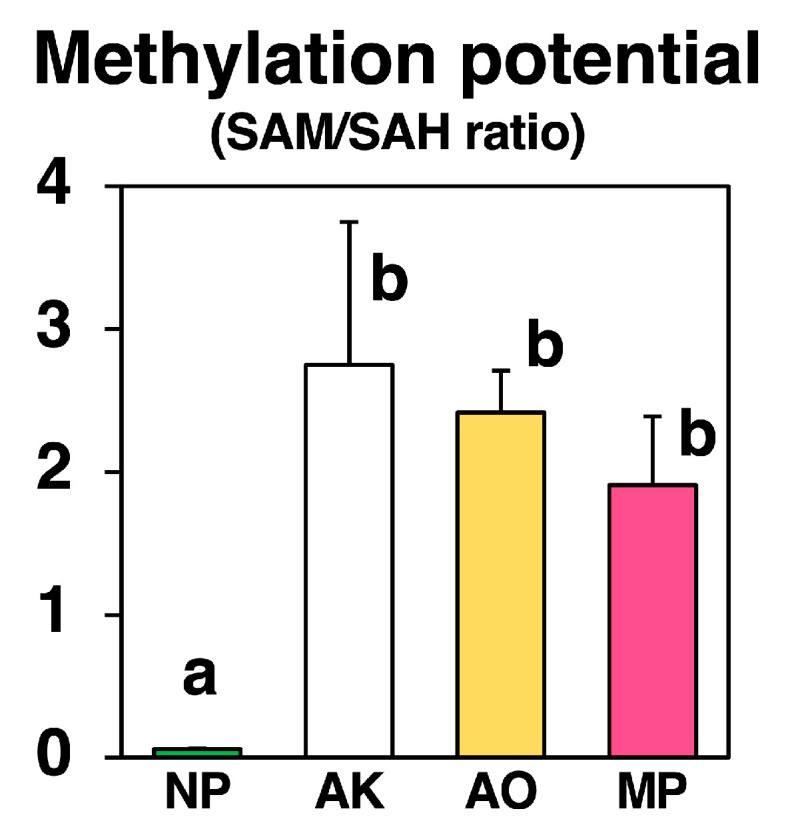
Ratios of *S*-adenosylmethionine (SAM) to *S*-adenosylhomocysteine (SAH) in non-fermented Nori (NP) and powders fermented with *Aspergillus oryzae* (AO), *A. luchuensis* mut. *kawachii* (AK), and *Monascus purpureus* (MP). Values are expressed as the mean ± standard error (n = 3 as biological replicates). Different letters on the bars denote significant differences (*p* < 0.05) among the groups.

To evaluate the cellular methylation environment during fermentation, we calculated the ratio of *S*-adenosylmethionine (SAM) to *S*-adenosylhomocysteine (SAH), a widely used indicator of methylation potential (Figure 7).

The SAM/SAH ratio, an indicator of methylation capacity, increased dramatically after fungal fermentation compared with that in non-fermented Nori. NP showed a low ratio of 0.060, whereas all *koji*-fermented samples showed markedly elevated ratios: 2.75 in AK, 2.42 in AO, and 1.91 in MP.

This shift indicates that *koji* fungal fermentation creates a methylation-favorable environment, likely activating multiple SAM-dependent amino acid methylation pathways.

## 3. Discussion

Many organisms, such as plants, bacteria, and fungi, engage in secondary metabolism, a process that generates non-essential compounds which enhance survival by providing various ecological benefits [15]. Fungi synthesize a wide array of secondary metabolites, some of which can be harmful to humans, while others, such as antibiotics, have been widely utilized for their therapeutic value [15,16]. We previously demonstrated that feeding of *koji*-fermented Nori powder exerts hepatoprotective effects in obese *db/db* mice and identified betaine structural analogs (betaine, stachydrine, and carnitine) as secondary metabolites present in the fermented product [7,8]. In this study, we conducted a comparative metabolomic analysis to examine the differences among *koji* fungus strains in the production of these betaine analogs and their precursors during seaweed fermentation. It is well established that secondary metabolite biosynthesis typically begins during the late phase of microbial growth and is closely associated with spore formation [8,15]. In our experiment, spore formation was observed after 72 h of fermentation using three different *koji* fungus strains (Figure 1).

Quaternary amines, such as betaine, stachydrine, and carnitine, are small nitrogen-containing molecules that carry a permanent positive charge, function as osmolytes by helping regulate intracellular water and salt balance, and also participate in methylation reactions [9,10,11,12,13,14]. Across the three *koji* fungi examined here, quaternary amine metabolism followed a consistent pattern with clear species-specific features. In all fermentations, increases in betaine and carnitine were observed, whereas stachydrine showed a pronounced strain hierarchy—high in *A. oryzae*, virtually absent in *A. kawachii*, and only trace amounts in *M. purpureus*.

The first is betaine (also called glycine betaine, Figure 4). All three fungi produced betaine *de novo* (it was not present in the starting material and appeared during fermentation), but they did so in different ways. *A. oryzae* produced betaine aldehyde, a short-lived intermediate that forms during the stepwise oxidation of choline to betaine. The detection of this intermediate provides direct evidence that the choline oxidation pathway (choline → betaine aldehyde → betaine) was active. This route aligns with reports from plant and microbial systems, where choline oxidation is catalyzed by either choline dehydrogenase/aldehyde dehydrogenase (ALDH) pairs or plant-type monooxygenase/ALDH systems [17,18]. *A. kawachii* exhibited a different behavior: choline levels dropped rapidly, no intermediate was detected, yet the final concentration of betaine was similar to that observed in *A. oryzae*. The simplest explanation is that the two oxidation steps are tightly coupled, preventing accumulation of the intermediate, or that a bifunctional choline oxidase catalyzes both steps within a single enzyme—both possibilities are known in bacteria and some fungi [19,20,21]. *M. purpureus* retained relatively high levels of choline but produced only moderate amounts of betaine. This suggests either a bottleneck in the choline oxidation pathway or that choline is diverted for other cellular functions. This interpretation aligns with the well-documented environmental regulation of secondary metabolism in *Monascus* species, which is influenced by factors such as nitrogen source, pH, temperature, and agitation. These factors can redirect enzymatic activity and gene expression away from specific pathways, such as stachydrine biosynthesis, while maintaining large precursor pools [22,23,24].

An alternative route—stepwise *N*-methylation of glycine to sarcosine, dimethylglycine, and finally to betaine—exists in some archaea and bacteria and is catalyzed by SAM-dependent methyltransferases (e.g., glycine/sarcosine *N*-methyltransferase). However, in our study, several lines of evidence suggest that this pathway played only a minor role, if any. *A. kawachii* accumulated high levels of betaine despite having a small glycine pool, and *A. oryzae* clearly showed the choline oxidation intermediate, both observations strongly support choline oxidation as the main source of betaine under our experimental conditions. Our previous research also supports this conclusion, as time-course metabolomic analysis of fermented seaweed using *A. oryzae* indicated that choline is the primary precursor of betaine biosynthesis [8]. While sarcosine and dimethylglycine were not target-analyzed in the assay and thus their potential contribution cannot be completely ruled out, the collective data support choline oxidation as the dominant route in these fungi. Overall, the relationships among precursors, intermediates, and products—combined with multivariate analysis—suggest that enzyme capacity and the coupling of reaction steps, rather than precursor abundance, are the primary determinants of betaine output.

The second is stachydrine (also called proline betaine, Figure 5). Here, species differences were striking. *A. oryzae* produced large amounts of stachydrine, *A. kawachii* produced virtually none, and *M. purpureus* produced only trace levels, despite maintaining a substantial proline pool—the amino acid precursor. This demonstrates that precursor availability alone is insufficient for stachydrine production. We therefore propose a simple “precursor plus methylation” model: output depends on both (i) the extent of glutamate conversion to proline via the Δ^1^-pyrroline-5-carboxylate reductase (P5CS/P5CR) pathway, commonly activated during stress or growth transitions [25,26,27], and (ii) the activity of methyltransferases that methylate proline into stachydrine using SAM as the methyl donor [28,29]. *A. oryzae* appears to be active on both fronts (strong proline supply and high methyltransferase activity), *A. kawachii* appears weak on both, and *M. purpureus* likely downregulates or deactivates the proline-to-stachydrine branch during fermentation. This finding aligns with the known environmental sensitivity of *Monascus* secondary metabolism [22,23,24]. Notably, although the SAM/SAH ratio increased in all fungi (Figure 7), only *A. oryzae* translated this methylation-favorable condition into high stachydrine production. This underscores that methyl donor abundance alone is insufficient without the appropriate enzymatic machinery and regulatory context [12,28,29].

The third is carnitine (also called lysine betaine, Figure 6). Carnitine levels increased across all species, but the increases did not correlate with free lysine levels. This is plausible because, in eukaryotes and many fungi, carnitine is not directly synthesized from free lysine. Instead, it is derived from TML, which is released during the breakdown of proteins containing methylated lysine residues. TML is then converted via a conserved four-step pathway to γ-butyrobetaine and subsequently to carnitine. This pathway has even been reconstructed in both yeast and bacteria using fungal genes [30,31], highlighting it as a general eukaryotic strategy rather than a species-specific anomaly. The elevated SAM/SAH ratios observed provide a straightforward link: when methylation is favored, more lysine residues on proteins become trimethylated, leading to greater TML production during protein turnover. This, in turn, fuels the carnitine biosynthesis pathway. This mechanism explains why carnitine levels increased across all three fungi, even though their free lysine levels varied.

In summary, the three *koji* fungi exhibit distinct metabolic strategies for betaine structural analogs biosynthesis during fermentation on seaweed substrates: *A. oryzae* demonstrates a robust integration of precursor flow and methylation capacity. It efficiently converts glutamate to proline and further methylates proline into stachydrine, while simultaneously oxidizing choline to glycine betaine. This dual-pathway activity highlights its metabolic versatility and potential for targeted enhancement in fermentation-based production systems. *A. kawachii*, although proficient in choline oxidation and capable of reaching betaine levels comparable to *A. oryzae*, lacks the enzymatic machinery or precursor availability necessary for stachydrine synthesis. This suggests a narrower metabolic focus, potentially limiting its utility in applications requiring diverse betaine derivatives. *M. purpureus* maintains high proline levels but downregulates the stachydrine pathway, likely due to environmental regulation of secondary metabolism. Its metabolic profile reflects the prioritization of other biosynthetic routes, which may be advantageous for producing alternative secondary metabolites but not for stachydrine accumulation.

These findings highlight the importance of precursor routing, methylation potential, and regulatory context in shaping osmolyte biosynthesis in filamentous fungi. The differential metabolic profiles observed among the fungi suggest that targeted manipulation of precursor supply and methylation pathways could enable precision fermentation strategies for the production of functional betaine structural analogs and related compounds. Furthermore, the observed increase in carnitine levels, independent of free lysine pools, implies a potential link between protein methylation dynamics and osmolyte biosynthesis, warranting further investigation into the methylation-mediated regulation in fungal systems.

## 4. Research Limitations and Future Directions

Although this study quantified a wide range of metabolites, not all intermediates could be measured, and metabolite levels were assessed only at the 72 h endpoint. These limitations highlight the need for future studies incorporating time-course and flux analyses. Measuring additional intermediates such as sarcosine, dimethylglycine, TML, and γ-butyrobetaine would strengthen evidence for the sequential steps in the glycine methylation and TML-mediated carnitine biosynthesis pathways. Complementary to this, transcript and protein profiling of key enzymes—including choline dehydrogenase or choline oxidase, betaine aldehyde dehydrogenase, SAM-dependent methyltransferases (for glycine and proline methylation), and γ-butyrobetaine hydroxylase or TML hydroxylase—would clarify how these pathways are regulated and activated. Isotope tracing using deuterium-, ^13^C-, or ^15^N-labeled choline, glycine, proline, and lysine can further confirm the roles of choline oxidation, glycine methylation, proline methylation, and the protein-derived lysine route to carnitine. Finally, perturbation studies targeting methylation balance, combined with standardized fermentation variables (nitrogen, pH, temperature, agitation), will help clarify control points and environment-dependent shifts in metabolite production in *Monascus*.

## 5. Materials and Methods

### 5.1. Sample Preparation

Nori sheets, a hot-air-dried form of the seaweed *Pyropia yezoensis*, were supplied by JA Saga (Saga, Japan) and shredded prior to fungal fermentation. Fungal fermentation of shredded Nori was performed according to the method described by Inoue et al. [8], with slight modifications. Suspensions of *Aspergillus luchuensis* mut. *kawachii* NBRC4308, *Aspergillus oryzae* L342, and *Monascus purpureus* NBRC4482 (1.65, 1.64, and 1.64 × 10^7^ spores/mL, respectively) were prepared, and 5 mL of each suspension was added to 5 g of the shredded Nori placed in an autoclaved Erlenmeyer flask. After thorough mixing, the flask was sealed with a sterilized sponge plug and incubated in a fermentation apparatus (PF110D, KNEAD Co., Ltd., Kanagawa, Japan) at 30.5 °C and greater than 90% relative humidity for three days. 72 h was selected based on previous experiments [8], which showed that spore formation and significant accumulation of betaine structural analogs occur at this time point. The fermented samples were subsequently freeze-dried, powdered using a grinder (TML162, Tescom Denki Co., Ltd., Tokyo, Japan), and stored at −80 °C until use.

### 5.2. Capillary Electrophoresis Mass Spectrometry (CE MS)

Sample powders (10 mg, n = 3 as biological replicates) were suspended in 500 µL of methanol containing 8 µM of internal standards (methionine sulfone for cation and camphor 10-sulfonic acid for anion analyses), 500 µL of chloroform and 200 µL of Milli-Q water, and subsequently centrifuged (20,400× *g*, 3 min, 4 °C). The upper layer of each solution was transferred to a 1.5 mL test tube, evaporated for 30 min using a centrifugal concentrator, and then separated into two layers. The upper layer was centrifugally filtered through a PALL Nanosep 3-kD cutoff filter (OD003C34, Nihon Pall Ltd., Tokyo, Japan) at 9100× *g* at 4 °C. Thereafter, the filtrate was dried using a centrifugal concentrator (SPD2010, Thermo Fisher Scientific K.K., Tokyo, Japan). The residue was dissolved in 20 µL of Milli-Q water containing 200 μM of internal standards (3-aminopyrrolidine for cation and trimesic acid for anion analyses). The CE MS system and conditions were set as described by Oikawa et al. [32,33]. All CE-time-of-flight (TOF) MS experiments were performed using an Agilent G7100A CE Instrument (Agilent Technologies, Santa Clara, CA, USA), Agilent G6224A TOF LC/MS system, Agilent 1200 Infinity series G1311C Quad Pump VL, and the G1603A Agilent CE-MS adapter and G1607A Agilent CE-electrospray ionization (ESI) MS sprayer kit. The G1601BA 3D-CE ChemStation software Ver. B.04.03 for CE and G3335-64002 MH Workstation were used. Separations were performed using a fused silica capillary (50 μm i.d. × 100 cm total length) filled with the following electrolytes; 1 M formic acid for cation analyses or with 20 mM ammonium formate (pH 10.0) for anion analyses as the electrolyte. The sample solutions were injected at 50 mbar for 15 s (15 nL). Prior to each run, the capillary was flushed with electrolyte for 5 min. The applied voltage was set to 30 kV. The capillary temperature was maintained at 20 °C, and the sample tray was cooled to below 4 °C. Approximately 50% (*v*/*v*) methanol/water containing 0.5 μM reserpine was delivered as the sheath liquid at a rate of 10 μL/min. ESI-TOF MS was conducted in the positive ion mode for cation analyses or in negative ion mode for anion analyses, and the capillary voltage was set at 4 kV. The flow rate of the heated dry nitrogen gas (heater temperature, 300 °C) was maintained at 10 psig. In the TOF MS, the fragmentor, skimmer and Oct RFV voltages were set at 110, 50, and 160 V for cation analyses and at 120, 60, and 220 V for anion analyses, respectively. Each acquired spectrum was automatically recalibrated using the reference masses of the reference standards. The methanol dimer ion ([2M + H]+, *m*/*z* = 65.0597) and reserpine ([M + H]+, *m*/*z* = 609.2806) for cation analyses or the formic acid dimer ion ([2M − H]−, *m*/*z* = 91.0037) and reserpine ([M − H]−, *m*/*z* = 607.2661) for anion analyses provided the lock mass for exact mass measurements. The exact mass data were acquired at a rate of 1.5 cycles/s in the range of 50–1,000 *m*/*z*. The CE-TOF MS in this study was operated at an approximate resolving power of 20,000 (FWHM), achieving mass accuracy within ± 2 ppm. Authentic reference standards were employed for the identification and absolute quantification of all metabolites. The peak areas of the standard compounds were monitored to verify consistent analytical sensitivity [32,33].

### 5.3. Statistical Analysis

Analyses were conducted in MetaboAnalyst 6.0 (https://www.metaboanalyst.ca/; accessed on 15 September 2025). Volcano plots were generated on z-scored data using Fold Change (FC) analysis (|log2 FC| > 1.0) and *t*-tests (*p* < 0.05); hierarchical clustering was performed on z-scored data using Ward’s method with Euclidean distance; PLS-DA was performed on raw (unscaled) data, and important features were ranked by VIP and model coefficients. No data transformation, missing-value imputation, or feature filtering was applied in any analysis. Data values are expressed as the mean ± standard error. To assess the differences among the four groups, data were analyzed using one-way analysis of variance (ANOVA), and all differences were analyzed using Tukey’s honest significant difference (HSD) post hoc test using the StatPlus:mac Pro Version 8.0.1 (AnalystSoft Inc., Walnut, CA, USA) software. Differences were considered statistically significant at *p* < 0.05.

## Figures and Tables

**Table 1:** The quantity-changes (QC, µmol/g) in metabolite levels relative to those in Nori powder (NP) after 72 h of fermentation by *Aspergillus luchuensis* mut. *kawachii* (AK)*, Aspergillus oryzae* (AO), *and Monascus purpureus* (MP). The color of the first row in each column indicates the color name of the *koji.* The top 30 from 227 metabolites are listed, and three betaine structural analogs are highlighted in yellow.

	Increase in AK vs. NP		Increase in AO vs. NP		Increase in MP vs. NP	
Rank	Metabolite	QC	Metabolite	QC	Metabolite	QC
1	Betaine	2.4447	Betaine	2.7545	Betaine	2.1415
2	Gentisate	0.7741	Valine	1.9028	Proline	1.4578
3	Malate	0.4943	Leucine	1.7107	Hypoxanthine	0.9107
4	Allantoin	0.3522	Stachydrine	1.4124	Phenylalanine	0.8310
5	Xanthine	0.2787	Hypoxanthine	1.2425	Xanthine	0.8295
6	Glycyl-L-leucine	0.2618	Proline	1.2401	Methionine	0.5831
7	GalNAc	0.2557	Glycine	1.2226	Valine	0.5791
8	GlcNAc	0.2120	4-Oxohexanoate	1.0085	Malate	0.5704
9	Carnitine	0.2041	Phenylalanine	1.0026	Tyrosine	0.5207
10	Guanosine	0.1907	Xanthine	0.8014	Succinate	0.4644
11	Fumarate	0.1895	Methionine	0.7816	Leucine	0.4166
12	UDP-GlcNAc	0.1662	Isoleucine	0.6693	Glycyl-L-leucine	0.3730
13	*N*-Cbz-Gly	0.1513	Lysine	0.6404	Lysine	0.3339
14	Hypoxanthine	0.1470	5-Oxohexanoate	0.5279	Allantoin	0.2689
15	UDP-GalNAc	0.1419	Tyrosine	0.4990	Carnitine	0.2238
16	Methionine	0.1144	Glycyl-L-leucine	0.4179	Tryptophan	0.2026
17	6-Hydroxynicotinate	0.1049	α-Ketoglutarate	0.3489	GalNAc	0.1552
18	X5P	0.1027	Succinate	0.3076	Fumarate	0.1489
19	Ribu5P	0.1000	Nicotinate	0.3029	Glucosamine	0.1384
20	3-Hydroxyisobutyrate	0.0763	GalNAc	0.2799	Prostaglandin E2	0.1320
21	Quinate	0.0748	4-Methyl-2-oxovalerate	0.2617	Galactosamine	0.1287
22	Ribose-5-phosphate	0.0640	Pyruvate	0.2544	GlcNAc	0.1287
23	Adenosine	0.0603	Prostaglandin E2	0.2345	Guanosine	0.1165
24	*trans*-Aconitate	0.0450	GlcNAc	0.2322	Nicotinate	0.0998
25	Citrate	0.0418	Serine	0.2032	Malonate	0.0909
26	Glucosamine	0.0402	Threonine	0.1975	4-Oxohexanoate	0.0883
27	Nicotinate	0.0381	Carnitine	0.1651	*N*6-Acetyllysine	0.0690
28	Galactosamine	0.0374	Histidine	0.1563	α-Ketoglutarate	0.0619
29	Isocitrate	0.0295	Guanosine	0.1491	3-Isopropylmalate	0.0589
30	Tryptophan	0.0283	Allantoin	0.1282	Mevalonate	0.0585

GalNAc: *N*-acetylgalactosamine, GlcNAc: *N*-acetylglucosamine, UDP: Uridine diphosphate, *N*-Cbz-Gly: *N*-Benzyloxycarbonylglycine, X5P: Xylulose-5-phosphate, Ribu5P: Ribulose-5-phosphate.

## Data Availability

The original contributions presented in this study are included in the article and supplementary material. Further inquiries can be directed to the corresponding author.

## Supplementary Materials

The following supporting information can be downloaded at: https://www.mdpi.com/article/10.3390/molecules30204104/s1, Figure S1: Venn diagram analysis of major metabolic alterations among *koji* fungi; Table S1: The quantity-changes (µmol/g) and fold changes in metabolite levels relative to those in Nori powder (NP) after 72 h of fermentation by *Aspergillus luchuensis* mut. *kawachii* (AK). 227 metabolites are listed, and three betaine structural analogs are highlighted in yellow; Table S2: The quantity-changes (µmol/g) and fold changes in metabolite levels relative to those in Nori powder (NP) after 72 h of fermentation by *Aspergillus oryzae* (AO). 227 metabolites are listed, and three betaine structural analogs are highlighted in yellow; Table S3: The quantity-changes (µmol/g) and fold changes in metabolite levels relative to those in Nori powder (NP) after 72 h of fermentation by *Monascus purpureus* (MP). 227 metabolites are listed, and three betaine structural analogs are highlighted in yellow.
