# Supplementary material for: Metabolomic Analysis of Fermented Nori Powders: Divergence of Betaine Structural Analogs Production by Three Types of koji Fungal Fermentation
Source: Molecules. 2025 Oct 16;30(20):4104. doi: 10.3390/molecules30204104 (PMC12566454; doi:10.3390/molecules30204104)
Supplement: Supplementary file 1 [file molecules-30-04104-s001.zip › molecules-3920905-supplementary.pdf]

# Supplementary Materials

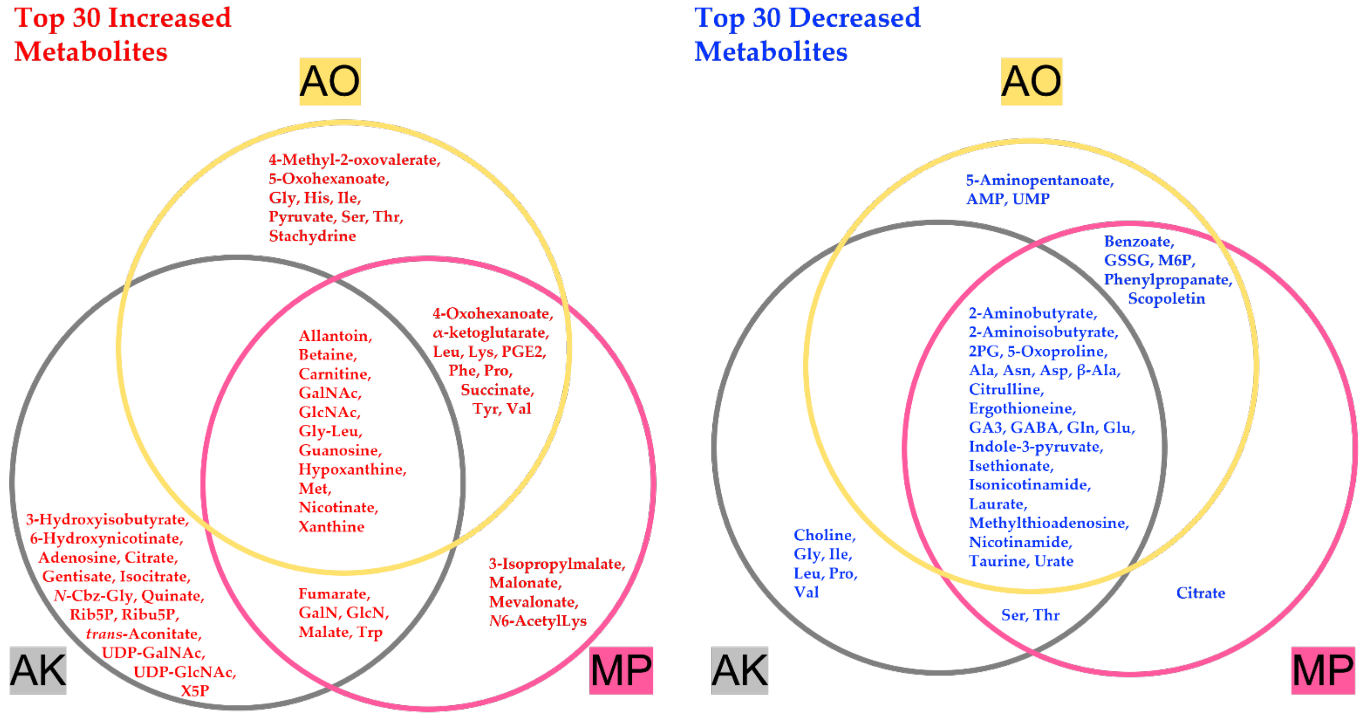


**Figure S1. Venn diagram analysis of major metabolic alterations among *koji* fungi.**

Venn diagrams showing the overlap of the top 30 metabolites with the greatest increases (left) and decreases (right) in quantity among *Aspergillus luchuensis* mut. *kawachii* (AK) vs Nori powder (NP), *Aspergillus oryzae* (AO) vs NP, and *Monascus purpureus* (MP) vs NP. The analysis highlights both shared and strain-specific metabolic shifts, based on ranked changes from the complete dataset (Tables S1–S3).

2PG: Glycerate 2-phosphate, Ala: Alanine, AMP: Adenosine monophosphate, Asn: Asparagine, Asp: Aspartic acid, β-Ala: β-Alanine, GA3: Gibberellic acid, GABA: γ-Aminobutyric acid, GalN: Galactosamine, GalNAc: *N*-acetylgalactosamine, GlcN: Glucosamine, GlcNAc: *N*-acetylglucosamine, Gln: Glutamine, Glu: Glutamic acid, Gly: Glycine, Gly-Leu: Glycyl-L-leucine, GSSG: Glutathione disulfide, His: Histidine, Ile: Isoleucine, Leu: Leucine, Lys: Lysine, M6P: Mannose-6-phosphate, Met: Methionine, *N*6-AcetylLys: *N*6-Acetyllysine, *N*-Cbz-Gly: *N*-Benzyloxycarbonylglycine, PGE2: Prostaglandin E2, Phe: Phenylalanine, Pro: Proline, Rib5P: Ribose-5-phosphate, Ribu5P: Ribulose-5-phosphate, Ser: Serine, Thr: Threonine, Trp: Tryptophan, Tyr: Tyrosine, UDP: Uridine diphosphate, UMP: Uridine monophosphate, Val: Valine, X5P: Xylulose-5-phosphate.

**Table S1.** The quantity-changes (μmol/g) and fold changes in metabolite levels relative to those in Nori powder (NP) after 72 h of fermentation by *Aspergillus luchuensis* mut. *kawachii* (AK). 227 metabolites are listed, and three betaine structural analogs are highlighted in yellow.

| | Changes in AK from NP | | |
|---|---|---|---|
| **Rank** | **Metabolite** | **Quantity change** | **Fold change** |
| 1 | Betaine | 2.4447 | Appear |
| 2 | Gentisate | 0.7741 | Appear |
| 3 | Malate | 0.4943 | 2.409 |
| 4 | Allantoin | 0.3522 | Appear |
| 5 | Xanthine | 0.2787 | Appear |
| 6 | Glycyl-L-leucine | 0.2618 | 29.820 |
| 7 | *N*-acetylgalactosamine | 0.2557 | 2.837 |
| 8 | *N*-acetylglucosamine | 0.2120 | 2.837 |
| 9 | Carnitine | 0.2041 | 364.964 |
| 10 | Guanosine | 0.1907 | 29.708 |
| 11 | Fumarate | 0.1895 | 4.705 |
| 12 | Uridine diphosphate-*N*-acetylglucosamine | 0.1662 | 13.757 |
| 13 | *N*-Benzyloxycarbonylglycine | 0.1513 | 11.450 |
| 14 | Hypoxanthine | 0.1470 | 141.747 |
| 15 | Uridine diphosphate-*N*-acetylgalactosamine | 0.1419 | 13.757 |
| 16 | Methionine | 0.1144 | 3.076 |
| 17 | 6-Hydroxynicotinate | 0.1049 | Appear |
| 18 | Xylulose-5-phosphate | 0.1027 | Appear |
| 19 | Ribulose-5-phosphate | 0.1000 | Appear |
| 20 | 3-Hydroxyisobutyrate | 0.0763 | 5.307 |
| 21 | Quinate | 0.0748 | 2.789 |
| 22 | Ribose-5-phosphate | 0.0640 | Appear |
| 23 | Adenosine | 0.0603 | 4.842 |
| 24 | *trans*-Aconitate | 0.0450 | 4.096 |
| 25 | Citrate | 0.0418 | 1.258 |
| 26 | Glucosamine | 0.0402 | Appear |
| 27 | Nicotinate | 0.0381 | 66.795 |
| 28 | Galactosamine | 0.0374 | Appear |
| 29 | Isocitrate | 0.0295 | Appear |
| 30 | Tryptophan | 0.0283 | 1.340 |
| 31 | Prostaglandin E2 | 0.0273 | 4.066 |
| 32 | Thidiazuron | 0.0256 | Appear |
| 33 | Malonate | 0.0253 | Appear |
| 34 | Adenine | 0.0202 | 5.779 |
| 35 | Pantothenate | 0.0199 | 5.331 |
| 36 | Mevalonate | 0.0189 | 3.104 |

| | | | |
|---|---|---|---|
| 37 | Raffinose | 0.0184 | 10.358 |
| 38 | 2-Aminosalicylate | 0.0179 | Appear |
| 39 | Inosine monophosphate | 0.0173 | Appear |
| 40 | Carboxybenzaldehyde | 0.0154 | Appear |
| 41 | Urocanate | 0.0140 | Appear |
| 42 | 3-Isopropylmalate | 0.0140 | Appear |
| 43 | *cis*-Aconitate | 0.0137 | 4.096 |
| 44 | Alanylalanine | 0.0134 | 110.010 |
| 45 | 5-Amino-4-oxovalerate | 0.0125 | 1.339 |
| 46 | $\alpha$-Ketoglutarate | 0.0119 | 2.718 |
| 47 | Hydroxyphenylacetate | 0.0095 | Appear |
| 48 | 3-Hydroxybutyrate | 0.0091 | 5.307 |
| 49 | Imidazole-4-acetate | 0.0083 | Appear |
| 50 | 2-Isopropylmalate | 0.0075 | Appear |
| 51 | Guanine | 0.0067 | 6.542 |
| 52 | Phenylacetylglycine | 0.0062 | Appear |
| 53 | Mevalonolactone | 0.0061 | 3.104 |
| 54 | Ornithine | 0.0060 | 1.505 |
| 55 | 4-Oxovalerate | 0.0059 | Appear |
| 56 | Thiamine | 0.0058 | Appear |
| 57 | 2-Aminoadipate | 0.0057 | 7.082 |
| 58 | Allantoate | 0.0057 | 2.434 |
| 59 | Trigonelline | 0.0053 | Appear |
| 60 | Anthranilate | 0.0053 | Appear |
| 61 | 2-Methylserine | 0.0051 | 2.319 |
| 62 | Pyridoxamine | 0.0051 | 17.628 |
| 63 | 6-Methyluracil | 0.0050 | Appear |
| 64 | 5-Methoxytryptamine | 0.0049 | Appear |
| 65 | Guanosine-3',5'-cyclic monophosphate | 0.0047 | 3.475 |
| 66 | Cytidine | 0.0043 | 1.335 |
| 67 | Tryptamine | 0.0041 | Appear |
| 68 | γ-Guanidinobutyrate | 0.0041 | Appear |
| 69 | *N*-acetylglucosamine 1-phosphate | 0.0040 | 18.129 |
| 70 | Saccharopine | 0.0037 | 3.160 |
| 71 | Cystathionine | 0.0037 | 2.845 |
| 72 | *N*-Acetyl-β-Alanine | 0.0036 | 1.339 |
| 73 | Citramalate | 0.0030 | 5.083 |
| 74 | Hexylamine | 0.0026 | Appear |
| 75 | *N*-Acetylleucine | 0.0022 | 4.258 |
| 76 | 3-Aminoisobutyrate | 0.0021 | Appear |
| 77 | Tauro-β-muricholate | 0.0018 | Appear |

| | | | |
|---|---|---|---|
| 78 | Hippurate | 0.0017 | Appear |
| 79 | Calmodulin-like protein | 0.0017 | Appear |
| 80 | Pyridoxal | 0.0017 | 2.756 |
| 81 | 5-Methylcytosine | 0.0015 | Appear |
| 82 | Stachydrine | 0.0015 | Appear |
| 83 | Guanosine monophosphate | 0.0013 | Appear |
| 84 | Deoxyguanosine | 0.0013 | 21.335 |
| 85 | Cystine | 0.0012 | 4.258 |
| 86 | Cytosine | 0.0011 | 2.379 |
| 87 | *N*-Acetylornithine | 0.0011 | 5.768 |
| 88 | β-Imidazolelactate | 0.0010 | 4.636 |
| 89 | *p*-Aminobenzoate | 0.0009 | Appear |
| 90 | Guanidinosuccinate | 0.0009 | Appear |
| 91 | *N*-acetylgalactosamine 6-phosphate | 0.0009 | 1.504 |
| 92 | Kyotorphin | 0.0008 | Appear |
| 93 | Cholate | 0.0007 | Appear |
| 94 | 3-Methyladenine | 0.0007 | 3.595 |
| 95 | Homotyrosine | 0.0007 | Appear |
| 96 | *N*-acetylglucosamine 6-phosphate | 0.0007 | 1.504 |
| 97 | Kynurenine | 0.0007 | 2.591 |
| 98 | *N*-Acetylmuramate | 0.0005 | 1.798 |
| 99 | 3-Aminobutyrate | 0.0005 | Appear |
| 100 | Thiamine diphosphate-glucose | 0.0004 | Appear |
| 101 | 2,4-Diaminobutanoate | 0.0004 | 5.288 |
| 102 | *N*8-Acetylspermidine | 0.0004 | Appear |
| 103 | Arginosuccinate | 0.0004 | 1.104 |
| 104 | *N*-Acetylaspartic acid | 0.0003 | 1.637 |
| 105 | Cytidine-3',5'-cyclic monophosphate | 0.0002 | 1.280 |
| 106 | 3-Hydroxy-3-methylglutaric acid | 0.0002 | 1.157 |
| 107 | Taurocholate | 0.0002 | Appear |
| 108 | 4-Methyl-5-thiazoleethanol | 0.0001 | 1.044 |
| 109 | 3-Iodotyrosine | 0.0001 | 1.253 |
| 110 | 1-Methyladenosine | 0.0000 | 1.009 |
| 111 | Betaine-aldehyde | 0.0000 | N.D. |
| 112 | 1,3-Diaminopropane | 0.0000 | N.D. |
| 113 | Pyruvate | 0.0000 | N.D. |
| 114 | 4-Coumarate | 0.0000 | N.D. |
| 115 | 2-Hydroxyisobutyrate | 0.0000 | N.D. |
| 116 | Deamino-Nicotinamide adenine dinucleotide | 0.0000 | N.D. |
| 117 | Xanthosine monophosphate | 0.0000 | N.D. |
| 118 | Adipate | 0.0000 | N.D. |

| | | | |
|---|---|---|---|
| 119 | Methionine sulfoxide | 0.0000 | N.D. |
| 120 | 2-Hydroxybutyrate | 0.0000 | N.D. |
| 121 | Deamido-Nicotinamide adenine dinucleotide | 0.0000 | N.D. |
| 122 | 3-Aminopropanediol | -0.0001 | 0.517 |
| 123 | Fructose-leucine | -0.0001 | 0.894 |
| 124 | L-Methionyl-L-methionine | -0.0002 | Disappear |
| 125 | *S*-adenosylmethionine | -0.0005 | 0.718 |
| 126 | Adenosine-3',5'-cyclic monophosphate | -0.0005 | 0.528 |
| 127 | 1-Deoxynojirimycin | -0.0006 | Disappear |
| 128 | L-Cysteine-glutathione disulfide | -0.0007 | 0.684 |
| 129 | 3-Methylhistidine | -0.0008 | 0.471 |
| 130 | Histidinol | -0.0008 | Disappear |
| 131 | Galacturonate | -0.0010 | 0.847 |
| 132 | Fructose-glutamic acid | -0.0012 | 0.952 |
| 133 | Deoxyadenosine 5'-triphosphate | -0.0016 | Disappear |
| 134 | *N*-Formylmethionine | -0.0019 | Disappear |
| 135 | Glucuronate | -0.0020 | 0.847 |
| 136 | Aspartame | -0.0020 | Disappear |
| 137 | 2,6-Diaminopimelate | -0.0026 | 0.651 |
| 138 | Octopine | -0.0028 | 0.036 |
| 139 | Guanosine diphosphate-glucose | -0.0029 | Disappear |
| 140 | 2-Aminopimelic acid | -0.0031 | 0.814 |
| 141 | Uridine diphosphate glucose | -0.0033 | 0.568 |
| 142 | Fructose-alanine | -0.0046 | 0.849 |
| 143 | Glutamylglutamine | -0.0047 | 0.176 |
| 144 | *N*6-Acetyllysine | -0.0049 | 0.727 |
| 145 | *N*-Acetylserine | -0.0050 | 0.449 |
| 146 | 6-Phospho-gluconate | -0.0059 | 0.537 |
| 147 | Diethanolamine | -0.0061 | 0.151 |
| 148 | Adenosine triphosphate | -0.0068 | Disappear |
| 149 | 4-Methyl-2-oxovalerate | -0.0071 | 0.223 |
| 150 | Isoasparagine | -0.0082 | 0.206 |
| 151 | Nicotinamide adenine dinucleotide | -0.0083 | 0.315 |
| 152 | Guanosine diphosphate | -0.0090 | 0.021 |
| 153 | Glucose-1-phosphate | -0.0102 | 0.260 |
| 154 | Sinapate | -0.0112 | 0.284 |
| 155 | Pipecolate | -0.0125 | 0.478 |
| 156 | Sedoheptulose 7-phosphate | -0.0141 | 0.065 |
| 157 | 5-Oxohexanoate | -0.0143 | 0.223 |
| 158 | Pyridoxamine 5-phosphate | -0.0145 | 0.115 |
| 159 | Uridine diphosphate-galactose | -0.0173 | 0.568 |

| | | | |
|---|---|---|---|
| 160 | Tyrosine | -0.0180 | 0.925 |
| 161 | Glutamyl-L-alanine | -0.0184 | 0.084 |
| 162 | Gluconate | -0.0192 | 0.317 |
| 163 | Inositol-1-phosphate | -0.0198 | 0.260 |
| 164 | Cytidine dinophosphate | -0.0226 | Disappear |
| 165 | Mannose-1-phosphate | -0.0226 | 0.260 |
| 166 | 4-Oxohexanoate | -0.0273 | 0.223 |
| 167 | *N*-Acetylglutamic acid | -0.0282 | 0.105 |
| 168 | Glycerate | -0.0283 | 0.281 |
| 169 | *S*-adenosylhomocysteine | -0.0306 | 0.015 |
| 170 | Cytidine monophosphate | -0.0317 | 0.317 |
| 171 | Glycerate 3-phosphate | -0.0328 | 0.310 |
| 172 | Glutathione | -0.0336 | 0.012 |
| 173 | Uridine diphosphate | -0.0353 | 0.015 |
| 174 | Indole-3-acetic acid | -0.0372 | 0.151 |
| 175 | Fructose-6-phosphate | -0.0379 | 0.134 |
| 176 | Galactose-1-phosphate | -0.0385 | 0.260 |
| 177 | Phenethylamine | -0.0407 | 0.089 |
| 178 | Adenosine diphosphate | -0.0431 | 0.089 |
| 179 | Phenylpyruvate | -0.0442 | 0.072 |
| 180 | Glucose-6-phosphate | -0.0478 | 0.134 |
| 181 | Arginine | -0.0479 | 0.736 |
| 182 | Glycerol-3-phosphate | -0.0544 | 0.249 |
| 183 | Oxamate | -0.0546 | 0.280 |
| 184 | Histidine | -0.0570 | 0.662 |
| 185 | Homoserine | -0.0603 | 0.044 |
| 186 | Lysine | -0.0700 | 0.621 |
| 187 | 5-Aminopentanoate | -0.0728 | 0.554 |
| 188 | Adenosine monophosphate | -0.0746 | 0.143 |
| 189 | Decanoate | -0.0761 | Disappear |
| 190 | Uridine monophosphate | -0.0961 | 0.113 |
| 191 | Scopoletin | -0.1018 | Disappear |
| 192 | Phenylpropanoate | -0.1124 | 0.285 |
| 193 | Mannose-6-phosphate | -0.1181 | 0.134 |
| 194 | Benzoate | -0.1421 | Disappear |
| 195 | Phenylalanine | -0.1562 | 0.650 |
| 196 | Glutathione disulfide | -0.1602 | 0.023 |
| 197 | Succinate | -0.1610 | 0.255 |
| 198 | 2-Aminoisobutyrate | -0.1982 | 0.060 |
| 199 | Glycerate 2-phosphate | -0.2047 | 0.310 |
| 200 | Methylthioadenosine | -0.2067 | 0.010 |

| | | | |
|---|---|---|---|
| 201 | Laurate | -0.2397 | Disappear |
| 202 | Proline | -0.2605 | 0.492 |
| 203 | 2-Aminobutyrate | -0.2705 | 0.060 |
| 204 | Isonicotinamide | -0.2827 | 0.017 |
| 205 | Nicotinamide | -0.2833 | 0.017 |
| 206 | Choline | -0.2904 | 0.342 |
| 207 | 5-Oxoproline | -0.3166 | 0.063 |
| 208 | Citrulline | -0.4008 | 0.063 |
| 209 | Glycine | -0.4843 | 0.137 |
| 210 | Isoleucine | -0.5037 | 0.212 |
| 211 | Glutamine | -0.5787 | 0.478 |
| 212 | Leucine | -0.6122 | 0.306 |
| 213 | Valine | -0.7859 | 0.305 |
| 214 | Ergothioneine | -0.8296 | 0.565 |
| 215 | β-Alanine | -0.8319 | 0.023 |
| 216 | Serine | -0.8727 | 0.087 |
| 217 | Threonine | -0.9873 | 0.074 |
| 218 | Indole-3-pyruvate | -1.0207 | 0.277 |
| 219 | γ-Aminobutyric acid | -1.4912 | 0.012 |
| 220 | Asparagine | -1.5502 | 0.050 |
| 221 | Gibberellic acid | -1.6591 | 0.489 |
| 222 | Isethionate | -2.1949 | 0.639 |
| 223 | Aspartic acid | -5.6720 | 0.025 |
| 224 | Taurine | -12.6853 | 0.532 |
| 225 | Glutamic acid | -14.7690 | 0.057 |
| 226 | Alanine | -38.8641 | 0.006 |
| 227 | Urate | -79.9669 | 0.355 |

**Table S2.** The quantity-changes (μmol/g) and fold changes in metabolite levels relative to those in Nori powder (NP) after 72 h of fermentation by *Aspergillus oryzae* (AO). 227 metabolites are listed, and three betaine structural analogs are highlighted in yellow.

| | Changes in AO from NP | | |
|---|---|---|---|
| **Rank** | **Metabolite** | **Quantity change** | **Fold change** |
| 1 | Betaine | 2.7545 | Appear |
| 2 | Valine | 1.9028 | 2.682 |
| 3 | Leucine | 1.7107 | 2.938 |
| 4 | Stachydrine | 1.4124 | Appear |
| 5 | Hypoxanthine | 1.2425 | 1190.963 |
| 6 | Proline | 1.2401 | 3.417 |
| 7 | Glycine | 1.2226 | 3.178 |
| 8 | 4-Oxohexanoate | 1.0085 | 29.738 |
| 9 | Phenylalanine | 1.0026 | 3.249 |
| 10 | Xanthine | 0.8014 | Appear |
| 11 | Methionine | 0.7816 | 15.184 |
| 12 | Isoleucine | 0.6693 | 2.047 |
| 13 | Lysine | 0.6404 | 4.468 |
| 14 | 5-Oxohexanoate | 0.5279 | 29.738 |
| 15 | Tyrosine | 0.4990 | 3.067 |
| 16 | Glycyl-L-leucine | 0.4179 | 46.994 |
| 17 | $\alpha$-Ketoglutarate | 0.3489 | 51.411 |
| 18 | Succinate | 0.3076 | 2.423 |
| 19 | Nicotinate | 0.3029 | 523.752 |
| 20 | *N*-acetylgalactosamine | 0.2799 | 3.011 |
| 21 | 4-Methyl-2-oxovalerate | 0.2617 | 29.738 |
| 22 | Pyruvate | 0.2544 | Appear |
| 23 | Prostaglandin E2 | 0.2345 | 27.384 |
| 24 | *N*-acetylglucosamine | 0.2322 | 3.011 |
| 25 | Serine | 0.2032 | 1.213 |
| 26 | Threonine | 0.1975 | 1.185 |
| 27 | Carnitine | 0.1651 | 295.288 |
| 28 | Histidine | 0.1563 | 1.927 |
| 29 | Guanosine | 0.1491 | 23.444 |
| 30 | Allantoin | 0.1282 | Appear |
| 31 | Tryptophan | 0.1181 | 2.420 |
| 32 | Ornithine | 0.1094 | 10.185 |
| 33 | Glucosamine | 0.0994 | Appear |
| 34 | Galactosamine | 0.0925 | Appear |
| 35 | Thidiazuron | 0.0886 | Appear |
| 36 | 5-Amino-4-oxovalerate | 0.0879 | 3.389 |
| 37 | Homoserine | 0.0842 | 2.335 |

| | | | |
|---|---|---|---|
| 38 | Inosine monophosphate | 0.0739 | Appear |
| 39 | Mevalonate | 0.0651 | 8.250 |
| 40 | 3-Hydroxyisobutyrate | 0.0630 | 4.556 |
| 41 | 2-Aminosalicylate | 0.0551 | Appear |
| 42 | *N*-Benzyloxycarbonylglycine | 0.0539 | 4.723 |
| 43 | Fumarate | 0.0518 | 2.013 |
| 44 | Malate | 0.0464 | 1.132 |
| 45 | Trigonelline | 0.0444 | Appear |
| 46 | Anthranilate | 0.0437 | Appear |
| 47 | Uridine diphosphate-*N*-acetylglucosamine | 0.0392 | 4.008 |
| 48 | Glycerate | 0.0364 | 1.924 |
| 49 | Malonate | 0.0360 | Appear |
| 50 | Gluconate | 0.0334 | 2.188 |
| 51 | Uridine diphosphate-*N*-acetylgalactosamine | 0.0334 | 4.008 |
| 52 | Betaine-aldehyde | 0.0317 | Appear |
| 53 | *N*6-Acetyllysine | 0.0302 | 2.681 |
| 54 | *N*-Acetyl-β-Alanine | 0.0254 | 3.389 |
| 55 | Guanosine-3',5'-cyclic monophosphate | 0.0234 | 13.410 |
| 56 | Mevalonolactone | 0.0211 | 8.250 |
| 57 | Arginine | 0.0209 | 1.115 |
| 58 | Hippurate | 0.0208 | Appear |
| 59 | Alanylalanine | 0.0208 | 170.473 |
| 60 | 3-Hydroxy-3-methylglutaric acid | 0.0192 | 18.579 |
| 61 | Methionine sulfoxide | 0.0177 | Appear |
| 62 | 3-Isopropylmalate | 0.0170 | Appear |
| 63 | Cystine | 0.0148 | 42.505 |
| 64 | Imidazole-4-acetate | 0.0143 | Appear |
| 65 | Hydroxyphenylacetate | 0.0136 | Appear |
| 66 | Isoasparagine | 0.0126 | 2.213 |
| 67 | 4-Oxovalerate | 0.0125 | Appear |
| 68 | γ-Guanidinobutyrate | 0.0122 | Appear |
| 69 | *N*-Acetylleucine | 0.0103 | 16.242 |
| 70 | 1,3-Diaminopropane | 0.0101 | Appear |
| 71 | 2-Isopropylmalate | 0.0091 | Appear |
| 72 | *N*-Acetylserine | 0.0081 | 1.900 |
| 73 | Fructose-alanine | 0.0080 | 1.261 |
| 74 | *trans*-Aconitate | 0.0078 | 1.539 |
| 75 | *N*-acetylglucosamine 1-phosphate | 0.0076 | 33.956 |
| 76 | Saccharopine | 0.0075 | 5.385 |
| 77 | 3-Hydroxybutyrate | 0.0075 | 4.556 |
| 78 | 2-Methylserine | 0.0070 | 2.805 |

| | | | |
|---|---|---|---|
| 79 | Fructose-glutamic acid | 0.0063 | 1.260 |
| 80 | Pyridoxal | 0.0060 | 7.291 |
| 81 | *N*-Acetylornithine | 0.0058 | 27.174 |
| 82 | Citramalate | 0.0058 | 8.962 |
| 83 | Cytidine-3',5'-cyclic monophosphate | 0.0055 | 7.216 |
| 84 | Pantothenate | 0.0055 | 2.197 |
| 85 | Raffinose | 0.0054 | 3.752 |
| 86 | Urocanate | 0.0052 | Appear |
| 87 | 2-Aminoadipate | 0.0051 | 6.413 |
| 88 | 6-Methyluracil | 0.0049 | Appear |
| 89 | Octopine | 0.0049 | 2.692 |
| 90 | 6-Phospho-gluconate | 0.0046 | 1.361 |
| 91 | Guanosine monophosphate | 0.0042 | Appear |
| 92 | 4-Coumarate | 0.0041 | Appear |
| 93 | Cytosine | 0.0039 | 5.738 |
| 94 | Arginosuccinate | 0.0039 | 2.056 |
| 95 | Allantoate | 0.0039 | 1.972 |
| 96 | 2-Hydroxyisobutyrate | 0.0039 | Appear |
| 97 | Homotyrosine | 0.0037 | Appear |
| 98 | Calmodulin-like protein | 0.0037 | Appear |
| 99 | Aspartame | 0.0035 | 2.713 |
| 100 | *p*-Aminobenzoate | 0.0034 | Appear |
| 101 | Guanine | 0.0034 | 3.808 |
| 102 | Adenine | 0.0034 | 1.796 |
| 103 | Isocitrate | 0.0034 | Appear |
| 104 | Glutamylglutamine | 0.0033 | 1.580 |
| 105 | Phenylacetylglycine | 0.0030 | Appear |
| 106 | Xanthosine monophosphate | 0.0029 | Appear |
| 107 | 5-Methoxytryptamine | 0.0028 | Appear |
| 108 | *N*-Formylmethionine | 0.0026 | 2.387 |
| 109 | *N*-Acetylaspartic acid | 0.0026 | 6.158 |
| 110 | 5-Methylcytosine | 0.0025 | Appear |
| 111 | Kyotorphin | 0.0024 | Appear |
| 112 | *cis*-Aconitate | 0.0024 | 1.539 |
| 113 | 3-Methyladenine | 0.0024 | 9.852 |
| 114 | Fructose-leucine | 0.0023 | 3.149 |
| 115 | L-Methionyl-L-methionine | 0.0021 | 14.627 |
| 116 | 2-Hydroxybutyrate | 0.0021 | Appear |
| 117 | Quinate | 0.0020 | 1.049 |
| 118 | Thiamine | 0.0020 | Appear |
| 119 | 3-Aminoisobutyrate | 0.0020 | Appear |

| | | | |
|---|---|---|---|
| 120 | Thiamine diphosphate-glucose | 0.0018 | Appear |
| 121 | Deoxyguanosine | 0.0017 | 28.747 |
| 122 | Pyridoxamine | 0.0015 | 5.932 |
| 123 | Guanidinosuccinate | 0.0015 | Appear |
| 124 | Tauro-β-muricholate | 0.0015 | Appear |
| 125 | *N*-Acetylmuramate | 0.0014 | 3.074 |
| 126 | *N*8-Acetylspermidine | 0.0012 | Appear |
| 127 | Adenosine-3',5'-cyclic monophosphate | 0.0012 | 2.063 |
| 128 | Cholate | 0.0012 | Appear |
| 129 | Kynurenine | 0.0011 | 3.724 |
| 130 | 3-Aminobutyrate | 0.0011 | Appear |
| 131 | 4-Methyl-5-thiazoleethanol | 0.0009 | 1.578 |
| 132 | 2,4-Diaminobutanoate | 0.0009 | 10.019 |
| 133 | Hexylamine | 0.0007 | Appear |
| 134 | β-Imidazolelactate | 0.0005 | 2.872 |
| 135 | 1-Deoxynojirimycin | 0.0004 | 1.696 |
| 136 | Cystathionine | 0.0004 | 1.186 |
| 137 | Histidinol | 0.0003 | 1.441 |
| 138 | 3-Iodotyrosine | 0.0002 | 1.706 |
| 139 | Taurocholate | 0.0001 | Appear |
| 140 | Tryptamine | 0.0001 | Appear |
| 141 | 2,6-Diaminopimelate | 0.0000 | 1.000 |
| 142 | 3-Aminopropanediol | 0.0000 | 0.834 |
| 143 | Ribulose-5-phosphate | 0.0000 | N.D. |
| 144 | Ribose-5-phosphate | 0.0000 | N.D. |
| 145 | Deamino-Nicotinamide adenine dinucleotide | 0.0000 | N.D. |
| 146 | Adipate | 0.0000 | N.D. |
| 147 | Xylulose-5-phosphate | 0.0000 | N.D. |
| 148 | Gentisate | 0.0000 | N.D. |
| 149 | 6-Hydroxynicotinate | 0.0000 | N.D. |
| 150 | Carboxybenzaldehyde | 0.0000 | N.D. |
| 151 | Deamido-Nicotinamide adenine dinucleotide | 0.0000 | N.D. |
| 152 | 1-Methyladenosine | -0.0001 | 0.712 |
| 153 | Galacturonate | -0.0004 | 0.936 |
| 154 | *S*-adenosylmethionine | -0.0005 | 0.720 |
| 155 | 3-Methylhistidine | -0.0005 | 0.640 |
| 156 | 2-Aminopimelic acid | -0.0006 | 0.966 |
| 157 | Glycerol-3-phosphate | -0.0006 | 0.991 |
| 158 | Glucuronate | -0.0008 | 0.936 |
| 159 | *N*-acetylglucosamine 6-phosphate | -0.0013 | Disappear |
| 160 | L-Cysteine-glutathione disulfide | -0.0016 | 0.239 |

| | | | |
|---|---|---|---|
| 161 | Deoxyadenosine 5'-triphosphate | -0.0016 | Disappear |
| 162 | *N*-acetylgalactosamine 6-phosphate | -0.0018 | Disappear |
| 163 | Glucose-1-phosphate | -0.0020 | 0.854 |
| 164 | Diethanolamine | -0.0025 | 0.657 |
| 165 | Guanosine diphosphate-glucose | -0.0029 | Disappear |
| 166 | Inositol-1-phosphate | -0.0039 | 0.854 |
| 167 | *N*-Acetylglutamic acid | -0.0042 | 0.866 |
| 168 | Mannose-1-phosphate | -0.0045 | 0.854 |
| 169 | Uridine diphosphate glucose | -0.0067 | 0.122 |
| 170 | Adenosine triphosphate | -0.0068 | Disappear |
| 171 | Glutamyl-L-alanine | -0.0068 | 0.660 |
| 172 | Galactose-1-phosphate | -0.0076 | 0.854 |
| 173 | Cytidine | -0.0091 | 0.296 |
| 174 | Guanosine diphosphate | -0.0092 | Disappear |
| 175 | Sinapate | -0.0101 | 0.354 |
| 176 | Nicotinamide adenine dinucleotide | -0.0111 | 0.087 |
| 177 | Sedoheptulose 7-phosphate | -0.0118 | 0.218 |
| 178 | Choline | -0.0119 | 0.973 |
| 179 | Adenosine | -0.0132 | 0.156 |
| 180 | Pyridoxamine 5-phosphate | -0.0147 | 0.104 |
| 181 | Pipecolate | -0.0163 | 0.320 |
| 182 | Phenylpyruvate | -0.0207 | 0.566 |
| 183 | Cytidine dinophosphate | -0.0226 | Disappear |
| 184 | Decanoate | -0.0235 | 0.691 |
| 185 | Fructose-6-phosphate | -0.0294 | 0.328 |
| 186 | *S*-adenosylhomocysteine | -0.0305 | 0.018 |
| 187 | Cytidine monophosphate | -0.0321 | 0.309 |
| 188 | Glutathione | -0.0335 | 0.015 |
| 189 | Uridine diphosphate-galactose | -0.0352 | 0.122 |
| 190 | Uridine diphosphate | -0.0358 | Disappear |
| 191 | Glycerate 3-phosphate | -0.0366 | 0.229 |
| 192 | Glucose-6-phosphate | -0.0371 | 0.328 |
| 193 | Indole-3-acetic acid | -0.0373 | 0.150 |
| 194 | Phenethylamine | -0.0404 | 0.096 |
| 195 | Adenosine diphosphate | -0.0464 | 0.021 |
| 196 | Citrate | -0.0516 | 0.681 |
| 197 | Oxamate | -0.0541 | 0.287 |
| 198 | 5-Aminopentanoate | -0.0603 | 0.630 |
| 199 | Phenylpropanoate | -0.0617 | 0.607 |
| 200 | Uridine monophosphate | -0.0844 | 0.221 |
| 201 | Adenosine monophosphate | -0.0847 | 0.027 |

| | | | |
|---|---|---|---|
| 202 | Benzoate | -0.0905 | 0.363 |
| 203 | Mannose-6-phosphate | -0.0916 | 0.328 |
| 204 | 2-Aminoisobutyrate | -0.0943 | 0.553 |
| 205 | Laurate | -0.0985 | 0.589 |
| 206 | Scopoletin | -0.1018 | Disappear |
| 207 | 2-Aminobutyrate | -0.1286 | 0.553 |
| 208 | Glutathione disulfide | -0.1640 | Disappear |
| 209 | 5-Oxoproline | -0.1720 | 0.491 |
| 210 | Indole-3-pyruvate | -0.2055 | 0.854 |
| 211 | Methylthioadenosine | -0.2062 | 0.012 |
| 212 | Glycerate 2-phosphate | -0.2287 | 0.229 |
| 213 | Citrulline | -0.2514 | 0.412 |
| 214 | Isonicotinamide | -0.2633 | 0.084 |
| 215 | Nicotinamide | -0.2639 | 0.084 |
| 216 | β-Alanine | -0.6804 | 0.201 |
| 217 | Ergothioneine | -0.8702 | 0.543 |
| 218 | Glutamine | -0.9664 | 0.129 |
| 219 | γ-Aminobutyric acid | -1.1432 | 0.243 |
| 220 | Gibberellic acid | -1.1813 | 0.636 |
| 221 | Isethionate | -1.2454 | 0.795 |
| 222 | Asparagine | -1.5351 | 0.059 |
| 223 | Aspartic acid | -5.4879 | 0.057 |
| 224 | Glutamic acid | -10.1967 | 0.349 |
| 225 | Taurine | -10.2335 | 0.623 |
| 226 | Alanine | -22.9596 | 0.413 |
| 227 | Urate | -65.4499 | 0.472 |

**Table S3.** The quantity-changes (μmol/g) and fold changes in metabolite levels relative to those in Nori powder (NP) after 72 h of fermentation by *Monascus purpureus* (MP). 227 metabolites are listed, and three betaine structural analogs are highlighted in yellow.

| | **Changes in MP from NP** | | |
|---|---|---|---|
| **Rank** | **Metabolite** | **Quantity change** | **Fold change** |
| 1 | Betaine | 2.1415 | Appear |
| 2 | Proline | 1.4578 | 3.841 |
| 3 | Hypoxanthine | 0.9107 | 873.214 |
| 4 | Phenylalanine | 0.8310 | 2.864 |
| 5 | Xanthine | 0.8295 | Appear |
| 6 | Methionine | 0.5831 | 11.581 |
| 7 | Valine | 0.5791 | 1.512 |
| 8 | Malate | 0.5704 | 2.626 |
| 9 | Tyrosine | 0.5207 | 3.157 |
| 10 | Succinate | 0.4644 | 3.148 |
| 11 | Leucine | 0.4166 | 1.472 |
| 12 | Glycyl-L-leucine | 0.3730 | 42.062 |
| 13 | Lysine | 0.3339 | 2.808 |
| 14 | Allantoin | 0.2689 | Appear |
| 15 | Carnitine | 0.2238 | 400.107 |
| 16 | Tryptophan | 0.2026 | 3.436 |
| 17 | *N*-acetylgalactosamine | 0.1552 | 2.115 |
| 18 | Fumarate | 0.1489 | 3.912 |
| 19 | Glucosamine | 0.1384 | Appear |
| 20 | Prostaglandin E2 | 0.1320 | 15.851 |
| 21 | Galactosamine | 0.1287 | Appear |
| 22 | *N*-acetylglucosamine | 0.1287 | 2.115 |
| 23 | Guanosine | 0.1165 | 18.530 |
| 24 | Nicotinate | 0.0998 | 173.281 |
| 25 | Malonate | 0.0909 | Appear |
| 26 | 4-Oxohexanoate | 0.0883 | 3.517 |
| 27 | *N*6-Acetyllysine | 0.0690 | 4.835 |
| 28 | $\alpha$-Ketoglutarate | 0.0619 | 9.950 |
| 29 | 3-Isopropylmalate | 0.0589 | Appear |
| 30 | Mevalonate | 0.0585 | 7.514 |
| 31 | Adipate | 0.0561 | Appear |
| 32 | 2-Aminosalicylate | 0.0559 | Appear |
| 33 | Gentisate | 0.0545 | Appear |
| 34 | Glycine | 0.0537 | 1.096 |
| 35 | Stachydrine | 0.0513 | Appear |
| 36 | 3-Hydroxyisobutyrate | 0.0513 | 3.898 |
| 37 | 5-Oxohexanoate | 0.0462 | 3.517 |

| | | | |
|---|---|---|---|
| 38 | Thidiazuron | 0.0460 | Appear |
| 39 | 6-Hydroxynicotinate | 0.0425 | Appear |
| 40 | Inosine monophosphate | 0.0424 | Appear |
| 41 | Ornithine | 0.0374 | 4.141 |
| 42 | Choline | 0.0351 | 1.080 |
| 43 | Pyruvate | 0.0350 | Appear |
| 44 | Guanine | 0.0337 | 29.036 |
| 45 | 5-Amino-4-oxovalerate | 0.0333 | 1.906 |
| 46 | 2-Isopropylmalate | 0.0315 | Appear |
| 47 | Deamido-Nicotinamide adenine dinucleotide | 0.0273 | Appear |
| 48 | Uridine diphosphate-*N*-acetylglucosamine | 0.0245 | 2.878 |
| 49 | 4-Methyl-2-oxovalerate | 0.0229 | 3.517 |
| 50 | Uridine diphosphate-*N*-acetylgalactosamine | 0.0209 | 2.878 |
| 51 | Mevalonolactone | 0.0190 | 7.514 |
| 52 | Tauro-β-muricholate | 0.0175 | Appear |
| 53 | 1,3-Diaminopropane | 0.0162 | Appear |
| 54 | Alanylalanine | 0.0158 | 129.673 |
| 55 | Urocanate | 0.0151 | Appear |
| 56 | Hydroxyphenylacetate | 0.0135 | Appear |
| 57 | 2-Methylserine | 0.0134 | 4.441 |
| 58 | *N*-Benzyloxycarbonylglycine | 0.0131 | 1.902 |
| 59 | Trigonelline | 0.0128 | Appear |
| 60 | Anthranilate | 0.0126 | Appear |
| 61 | Methionine sulfoxide | 0.0120 | Appear |
| 62 | 6-Methyluracil | 0.0115 | Appear |
| 63 | Gluconate | 0.0112 | 1.399 |
| 64 | Pantothenate | 0.0109 | 3.379 |
| 65 | Saccharopine | 0.0102 | 6.951 |
| 66 | Histidine | 0.0102 | 1.061 |
| 67 | Allantoate | 0.0101 | 3.525 |
| 68 | *N*-Acetyl-β-Alanine | 0.0096 | 1.906 |
| 69 | 3-Aminoisobutyrate | 0.0095 | Appear |
| 70 | Arginosuccinate | 0.0094 | 3.559 |
| 71 | 5-Methoxytryptamine | 0.0077 | Appear |
| 72 | Raffinose | 0.0077 | 4.908 |
| 73 | Imidazole-4-acetate | 0.0076 | Appear |
| 74 | Hippurate | 0.0071 | Appear |
| 75 | 4-Oxovalerate | 0.0069 | Appear |
| 76 | 2-Aminoadipate | 0.0068 | 8.149 |
| 77 | 3-Hydroxybutyrate | 0.0061 | 3.898 |
| 78 | Cytosine | 0.0060 | 8.288 |

| | | | |
|---|---|---|---|
| 79 | Pyridoxamine | 0.0056 | 19.409 |
| 80 | Cystathionine | 0.0055 | 3.779 |
| 81 | γ-Guanidinobutyrate | 0.0052 | Appear |
| 82 | Citramalate | 0.0044 | 7.089 |
| 83 | *N*-acetylglucosamine 1-phosphate | 0.0040 | 18.087 |
| 84 | Tryptamine | 0.0039 | Appear |
| 85 | Fructose-glutamic acid | 0.0039 | 1.159 |
| 86 | *N*-Acetylleucine | 0.0035 | 6.119 |
| 87 | 2-Hydroxyisobutyrate | 0.0033 | Appear |
| 88 | Cystine | 0.0033 | 10.300 |
| 89 | Homotyrosine | 0.0032 | Appear |
| 90 | Cholate | 0.0031 | Appear |
| 91 | Xanthosine monophosphate | 0.0031 | Appear |
| 92 | Deamino-Nicotinamide adenine dinucleotide | 0.0030 | Appear |
| 93 | *N*-Acetylornithine | 0.0030 | 14.615 |
| 94 | Pyridoxal | 0.0028 | 3.957 |
| 95 | Carboxybenzaldehyde | 0.0027 | Appear |
| 96 | Thiamine | 0.0026 | Appear |
| 97 | *p*-Aminobenzoate | 0.0026 | Appear |
| 98 | Guanosine-3',5'-cyclic monophosphate | 0.0023 | 2.221 |
| 99 | Guanosine monophosphate | 0.0022 | Appear |
| 100 | Calmodulin-like protein | 0.0020 | Appear |
| 101 | 3-Methyladenine | 0.0019 | 8.103 |
| 102 | 2-Hydroxybutyrate | 0.0018 | Appear |
| 103 | 5-Methylcytosine | 0.0018 | Appear |
| 104 | Taurocholate | 0.0017 | Appear |
| 105 | Fructose-leucine | 0.0016 | 2.555 |
| 106 | Kynurenine | 0.0016 | 4.773 |
| 107 | Deoxyguanosine | 0.0015 | 24.136 |
| 108 | 3-Aminobutyrate | 0.0014 | Appear |
| 109 | *N*-Formylmethionine | 0.0013 | 1.701 |
| 110 | Adenine | 0.0013 | 1.310 |
| 111 | *N*-Acetylserine | 0.0012 | 1.138 |
| 112 | Phenylacetylglycine | 0.0012 | Appear |
| 113 | *N*-Acetylaspartic acid | 0.0012 | 3.317 |
| 114 | *N*-Acetylmuramate | 0.0011 | 2.642 |
| 115 | β-Imidazolelactate | 0.0010 | 4.608 |
| 116 | 2,4-Diaminobutanoate | 0.0010 | 11.504 |
| 117 | Thiamine diphosphate-glucose | 0.0010 | Appear |
| 118 | Hexylamine | 0.0010 | Appear |
| 119 | Fructose-alanine | 0.0008 | 1.027 |

| | | | |
|---|---|---|---|
| 120 | Kyotorphin | 0.0008 | Appear |
| 121 | 1-Deoxynojirimycin | 0.0007 | 2.254 |
| 122 | Cytidine-3',5'-cyclic monophosphate | 0.0007 | 1.778 |
| 123 | Glycerate | 0.0005 | 1.014 |
| 124 | 3-Iodotyrosine | 0.0005 | 3.226 |
| 125 | 2,6-Diaminopimelate | 0.0004 | 1.055 |
| 126 | Diethanolamine | 0.0004 | 1.051 |
| 127 | 3-Hydroxy-3-methylglutaric acid | 0.0002 | 1.207 |
| 128 | Octopine | 0.0002 | 1.076 |
| 129 | Adenosine-3',5'-cyclic monophosphate | 0.0002 | 1.146 |
| 130 | *N*8-Acetylspermidine | 0.0002 | Appear |
| 131 | L-Methionyl-L-methionine | 0.0001 | 1.541 |
| 132 | 3-Aminopropanediol | 0.0000 | 0.984 |
| 133 | 4-Methyl-5-thiazoleethanol | 0.0000 | 0.983 |
| 134 | Betaine-aldehyde | 0.0000 | N.D. |
| 135 | Guanidinosuccinate | 0.0000 | N.D. |
| 136 | Isocitrate | 0.0000 | N.D. |
| 137 | Ribulose-5-phosphate | 0.0000 | N.D. |
| 138 | Ribose-5-phosphate | 0.0000 | N.D. |
| 139 | 4-Coumarate | 0.0000 | N.D. |
| 140 | Xylulose-5-phosphate | 0.0000 | N.D. |
| 141 | 3-Methylhistidine | -0.0001 | 0.935 |
| 142 | 1-Methyladenosine | -0.0003 | 0.386 |
| 143 | Aspartame | -0.0004 | 0.807 |
| 144 | Histidinol | -0.0005 | 0.313 |
| 145 | *N*-acetylglucosamine 6-phosphate | -0.0011 | 0.184 |
| 146 | L-Cysteine-glutathione disulfide | -0.0013 | 0.371 |
| 147 | *S*-adenosylmethionine | -0.0013 | 0.289 |
| 148 | *cis*-Aconitate | -0.0014 | 0.682 |
| 149 | *N*-acetylgalactosamine 6-phosphate | -0.0014 | 0.184 |
| 150 | Guanosine diphosphate-glucose | -0.0015 | 0.478 |
| 151 | Galacturonate | -0.0015 | 0.769 |
| 152 | Deoxyadenosine 5'-triphosphate | -0.0016 | Disappear |
| 153 | Glutamylglutamine | -0.0021 | 0.638 |
| 154 | Glucuronate | -0.0030 | 0.769 |
| 155 | 2-Aminopimelic acid | -0.0033 | 0.803 |
| 156 | 6-Phospho-gluconate | -0.0034 | 0.735 |
| 157 | Isoasparagine | -0.0041 | 0.601 |
| 158 | *trans*-Aconitate | -0.0046 | 0.682 |
| 159 | Uridine diphosphate glucose | -0.0066 | 0.134 |
| 160 | Adenosine triphosphate | -0.0068 | Disappear |

| | | | |
|---|---|---|---|
| 161 | Cytidine | -0.0074 | 0.426 |
| 162 | Pipecolate | -0.0078 | 0.673 |
| 163 | Glucose-1-phosphate | -0.0089 | 0.352 |
| 164 | Guanosine diphosphate | -0.0092 | Disappear |
| 165 | Nicotinamide adenine dinucleotide | -0.0098 | 0.192 |
| 166 | Glutamyl-L-alanine | -0.0103 | 0.486 |
| 167 | Sedoheptulose 7-phosphate | -0.0112 | 0.254 |
| 168 | Sinapate | -0.0113 | 0.279 |
| 169 | Adenosine | -0.0121 | 0.229 |
| 170 | Pyridoxamine 5-phosphate | -0.0139 | 0.151 |
| 171 | Inositol-1-phosphate | -0.0174 | 0.352 |
| 172 | Glycerol-3-phosphate | -0.0196 | 0.730 |
| 173 | Mannose-1-phosphate | -0.0198 | 0.352 |
| 174 | *N*-Acetylglutamic acid | -0.0202 | 0.358 |
| 175 | Cytidine dinophosphate | -0.0226 | Disappear |
| 176 | Homoserine | -0.0263 | 0.583 |
| 177 | *S*-adenosylhomocysteine | -0.0307 | 0.009 |
| 178 | Phenylpyruvate | -0.0322 | 0.325 |
| 179 | Glutathione | -0.0330 | 0.031 |
| 180 | Galactose-1-phosphate | -0.0337 | 0.352 |
| 181 | Uridine diphosphate-galactose | -0.0347 | 0.134 |
| 182 | Quinate | -0.0355 | 0.149 |
| 183 | Uridine diphosphate | -0.0358 | Disappear |
| 184 | Cytidine monophosphate | -0.0368 | 0.207 |
| 185 | Indole-3-acetic acid | -0.0373 | 0.150 |
| 186 | Fructose-6-phosphate | -0.0374 | 0.145 |
| 187 | Phenethylamine | -0.0382 | 0.145 |
| 188 | Glycerate 3-phosphate | -0.0391 | 0.177 |
| 189 | Adenosine diphosphate | -0.0456 | 0.037 |
| 190 | Glucose-6-phosphate | -0.0472 | 0.145 |
| 191 | 5-Aminopentanoate | -0.0493 | 0.698 |
| 192 | Oxamate | -0.0595 | 0.215 |
| 193 | Decanoate | -0.0761 | Disappear |
| 194 | Adenosine monophosphate | -0.0836 | 0.040 |
| 195 | Isoleucine | -0.0852 | 0.867 |
| 196 | Uridine monophosphate | -0.0897 | 0.173 |
| 197 | Arginine | -0.0944 | 0.481 |
| 198 | Citrate | -0.1012 | 0.374 |
| 199 | Scopoletin | -0.1018 | Disappear |
| 200 | Phenylpropanoate | -0.1048 | 0.333 |
| 201 | Mannose-6-phosphate | -0.1166 | 0.145 |

| | | | |
|---|---|---|---|
| 202 | Benzoate | -0.1262 | 0.112 |
| 203 | 2-Aminoisobutyrate | -0.1532 | 0.273 |
| 204 | Glutathione disulfide | -0.1620 | 0.012 |
| 205 | Methylthioadenosine | -0.2081 | 0.004 |
| 206 | 2-Aminobutyrate | -0.2090 | 0.273 |
| 207 | Laurate | -0.2369 | 0.011 |
| 208 | Glycerate 2-phosphate | -0.2441 | 0.177 |
| 209 | 5-Oxoproline | -0.2578 | 0.237 |
| 210 | Citrulline | -0.2720 | 0.364 |
| 211 | Isonicotinamide | -0.2761 | 0.040 |
| 212 | Nicotinamide | -0.2767 | 0.040 |
| 213 | Glutamine | -0.5961 | 0.462 |
| 214 | β-Alanine | -0.6177 | 0.275 |
| 215 | Serine | -0.6285 | 0.343 |
| 216 | Indole-3-pyruvate | -0.6738 | 0.522 |
| 217 | Threonine | -0.7079 | 0.336 |
| 218 | Ergothioneine | -0.9894 | 0.481 |
| 219 | γ-Aminobutyric acid | -1.2215 | 0.191 |
| 220 | Gibberellic acid | -1.4753 | 0.545 |
| 221 | Asparagine | -1.4760 | 0.095 |
| 222 | Isethionate | -2.6309 | 0.567 |
| 223 | Aspartic acid | -5.3127 | 0.087 |
| 224 | Glutamic acid | -11.4451 | 0.270 |
| 225 | Taurine | -13.0732 | 0.518 |
| 226 | Alanine | -29.6740 | 0.241 |
| 227 | Urate | -76.3155 | 0.384 |
